# Aggregate-prone brain regions in Parkinson’s disease are rich in unique N-terminus α-synuclein conformers with high proteolysis susceptibility

**DOI:** 10.1038/s41531-023-00614-w

**Published:** 2024-01-02

**Authors:** James A. Wiseman, Helen C. Murray, Richard L. M. F. Faull, Michael Dragunow, Clinton P. Turner, Birger Victor Dieriks, Maurice A. Curtis

**Affiliations:** 1 Department of Anatomy and Medical Imaging, University of Auckland, Auckland, New Zealand; 2 Centre for Brain Research, University of Auckland, Auckland, 1023 New Zealand; 3 Department of Pharmacology, University of Auckland, Auckland, 1023 New Zealand; 4 LabPlus, Department of Anatomical Pathology, Te Whatu Ora, Auckland, New Zealand

**Keywords:** Parkinson's disease, Brain, Microscopy, Parkinson's disease, Parkinson's disease

## Abstract

In Parkinson’s disease (PD), and other α-synucleinopathies, α-synuclein (α-Syn) aggregates form a myriad of conformational and truncational variants. Most antibodies used to detect and quantify α-Syn in the human brain target epitopes within the C-terminus (residues 96–140) of the 140 amino acid protein and may fail to capture the diversity of α-Syn variants present in PD. We sought to investigate the heterogeneity of α-Syn conformations and aggregation states in the PD human brain by labelling with multiple antibodies that detect epitopes along the entire length of α-Syn. We used multiplex immunohistochemistry to simultaneously immunolabel tissue sections with antibodies mapping the three structural domains of α-Syn. Discrete epitope-specific immunoreactivities were visualised and quantified in the olfactory bulb, medulla, substantia nigra, hippocampus, entorhinal cortex, middle temporal gyrus, and middle frontal gyrus of ten PD cases, and the middle temporal gyrus of 23 PD, and 24 neurologically normal cases. Distinct Lewy neurite and Lewy body aggregate morphologies were detected across all interrogated regions/cases. Lewy neurites were the most prominent in the olfactory bulb and hippocampus, while the substantia nigra, medulla and cortical regions showed a mixture of Lewy neurites and Lewy bodies. Importantly, unique N-terminus immunoreactivity revealed previously uncharacterised populations of (1) perinuclear, (2) glial (microglial and astrocytic), and (3) neuronal lysosomal α-Syn aggregates. These epitope-specific N-terminus immunoreactive aggregate populations were susceptible to proteolysis via time-dependent proteinase K digestion, suggesting a less stable oligomeric aggregation state. Our identification of unique N-terminus immunoreactive α-Syn aggregates adds to the emerging paradigm that α-Syn pathology is more abundant and complex in human brains with PD than previously realised. Our findings highlight that labelling multiple regions of the α-Syn protein is necessary to investigate the full spectrum of α-Syn pathology and prompt further investigation into the functional role of these N-terminus polymorphs.

## Introduction

α-Syn aggregation is the major neuropathological hallmark of several neurodegenerative diseases known as α-synucleinopathies, including Parkinson’s disease (PD), dementia with Lewy bodies, and multiple system atrophy^1–7^. In the healthy human central nervous system, α-Syn expression is abundant and predominantly localised to presynaptic nerve terminals where it plays a key role in synaptic vesicle trafficking, neurotransmission and synaptic plasticity^1,6,8,9^. Pathogenic misfolding and aggregation of α-Syn lead to the formation of insoluble intracellular aggregates called Lewy bodies (LBs) and Lewy neurites (LNs). While α-Syn is the common protein underlying these diseases, recent evidence suggests the α-Syn proteins within these aggregates carry a range of different post-translational modifications (PTMs)^1,6,9–15^. Profiling the structure of α-Syn is a necessary first step toward understanding the link between pathology, disease progression and symptom heterogeneity in α-synucleinopathies.

**Fig. 1:**
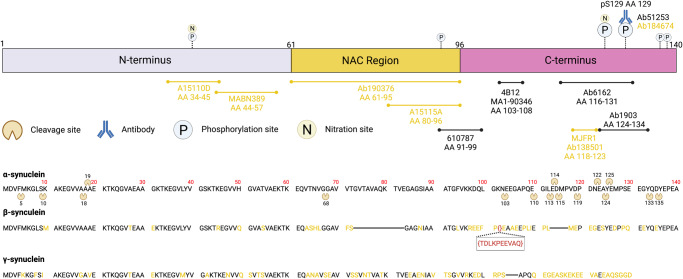
Schematic diagram depicting the amino acid sequence and three structural domains of the α-Syn protein. The common phosphorylation, nitration and truncation sites of α-Syn are also illustrated, along with a direct comparison between the respective BLAST amino acid sequences for α-Syn, β-synuclein and γ-synuclein. Yellow text in the β/γ-synuclein BLAST amino acid sequences denotes amino acid residues that are distinct from their corresponding α-Syn residue. Finally, an antibody-epitope map of the eleven epitope-specific α-Syn antibodies used in this study is shown. Antibodies used in the final optimised antibody panel are highlighted in yellow text. Created with BioRender.com.

Examination of α-Syn aggregates in human post-mortem brain tissue relies on the use of antibodies. Currently, most commercially available α-Syn antibodies detect epitopes within the C-terminus (Fig. 1), with only a select few identifying epitopes within the N-terminus or non-amyloid component (NAC) structural domains^16^. The negatively charged and hydrophilic C-terminus domain of α-Syn (residues 96–140) has no structure-forming propensity but encompasses amino acid residues that are associated with several PTMs (Fig. 1)^1,6,9–14^. Approximately 90% of the α-Syn deposited in LBs and LNs is phosphorylated at the serine 129 (pS129) residue within the C-terminal region; in contrast, only 4% of monomeric α-Syn is phosphorylated^17–19^. Therefore, pS129-specific α-Syn antibodies are widely used as the gold standard for identifying pathological α-Syn in post-mortem human brain tissue, as well as in cellular and animal models of α-synucleinopathies^12^.

The C-terminus also has several pathologically significant truncation sites (Fig. 1). Growing evidence suggests that C-terminally truncated variants of α-Syn significantly potentiate the propensity for α-Syn aggregation and promote its toxicity^2,20–24^. Two of the most consistently identified truncated α-Syn variants in human brain tissue are 1–119 (truncated at Asp-119) and 1–122 (truncated at Asn-122), which have an abundance as high as 20–25% relative to full-length α-Syn^18,20,25^. Accordingly, many α-Syn antibodies that identify epitopes within the C-terminus will fail to capture these pathologically significant variants of C-terminally truncated α-Syn^16^.

Zhang et al. reported that C-terminal truncations conformationally alter α-Syn by disrupting the long-range intramolecular interaction between the C-terminus and the N-terminus that typically maintain α-Syn in a relatively compact hairpin conformation^24^. Truncation-induced disruption of these intramolecular interactions causes α-Syn to adopt an elongated conformation, increasing the exposure of both the N-terminus and the amyloidogenic NAC region^24^. This increased exposure of the N-terminus permits stronger interaction of C-terminally-truncated α-Syn with membranes and molecular chaperones, resulting in increased mitochondrial and other cellular dysfunction^24^. In contrast to C-terminus truncations, McGlinchey et al. reported that N-terminally truncated α-Syn polymorphs are poor seeds for the aggregation of full-length α-Syn. Through a series of cross-fibril propagation experiments, they demonstrated that the N-terminus plays a vital role in α-Syn fibril formation and conformational structure, underscoring the importance of accurately detecting and investigating this pathologically significant α-Syn domain^26^.

Independent of these PTMs and truncational variants, α-Syn also exhibits remarkable chameleonic protein plasticity, which permits its microenvironment-dependent aggregation and ability to adopt a myriad of tertiary conformations^27–29^. This additional conformational heterogeneity potentiates the possibility for conformation-specific masking of certain α-Syn epitopes. The extent to which this occurs in human α-synucleinopathies, however, remains unknown and poorly studied.

Several studies have interrogated the unique staining profiles of both PTM- and epitope-specific α-Syn antibodies^15,25,30–32^, however, studies of non-C-terminus epitopes are still largely lacking. Of these studies, the N-terminus antibody, 5G4, has been shown to maintain a high affinity for the high-molecular weight fraction of β-sheet rich oligomers^31^, facilitating its preferential immunolabelling of pathological α-Syn^15,32^. Altay et al. also recently demonstrated that antibodies directed against the mid-late N-terminus (LASH-BL 34–45, 5G4) and late NAC domain (LASH-BL 80–96) detect astrocytic α-Syn within the human brain, which is not labelled with other canonical α-Syn antibodies^15^. Finally, Moors et al. utilised PTM- and epitope-specific α-Syn antibodies to characterise the unique arrangement of different α-Syn variants within nigral LBs^25^. In particular, they highlighted the condensed onion-like structure of nigral LBs, within which C-terminally-truncated (119CTT, 122CTT) α-Syn variants, and N-terminus and NAC epitopes are typically condensed within the LB core, with C-terminus epitopes and pS129 α-Syn predominantly localised to the LB periphery^25^.

Despite the inherent complexity and conformational variability of α-Syn in the human brain, it is common practice in the field to rely on single-epitope immunolabelling methods^33–35^. Capturing the conformational heterogeneity of α-Syn, instead, necessitates the detection of multiple epitopes in the same sample. In the present study, we validate and use epitope-specific antibodies to detect and quantify the three structural domains of α-Syn in different regions of the human brain with PD. Co-labelling with diverse α-Syn antibodies revealed that a substantial quantity of α-Syn is uniquely labelled only by antibodies targeted to the N-terminus (residues 34–45 and residues 44–57), the late NAC (80–96) domain, or to the pS129 PTM. Given the importance of the N-terminus in α-Syn fibril formation and the use of pS129-specific α-Syn antibodies in pathological classifications, our findings demonstrate the necessity of utilising epitope-specific multiplex immunohistochemistry to capture the diversity of α-Syn variants and the extent of pathology in α-synucleinopathies.

## Results

### Epitope-specific α-synuclein immunoreactivity in the human brain

To identify and quantify immunolabelling of the three structural domains of α-Syn in human PD brains (Table 1), we compiled a set of eleven commercially available epitope-specific antibodies (Fig. 1, Table 2). Systematic validations of different epitope-specific antibody combinations were conducted using multiplex fluorescence immunohistochemistry to establish an antibody panel that detected α-Syn epitopes in each structural domain and S129 phosphorylation, the most studied PTM. Under optimised antigen retrieval methods, all α-Syn antibodies detected α-Syn pathology in all brain regions. The final antibody panel was selected to maximise coverage of the α-Syn protein in pathological aggregates, whilst still ensuring species/isotype multiplex compatibility. For the N-terminus, 849102 (A15110D) specifically detects the amino acid residues 34–45, and MABN389 (5G4) detects residues 44–57. Ab190376 detects the entire NAC domain (residues 61–95), and 848302 (A15115A) detects residues 80–96 of the late NAC domain. For the C-terminus, Ab184674 (pS129, 81 A) specifically detects α-Syn that is phosphorylated at the serine 129 residue, and Ab138501 (MJFR1) specifically detects residues 118–123. Due to species/isotype overlap, we could not include MABN389 (5G4) and 848302 (A15115A) as part of the final multiplex antibody panel. Due to the high sequence homology of α-Syn with both β-synuclein and γ-synuclein, particularly within the N-terminus, the α-Syn-specificity of each of these candidate antibodies was validated by determining that there was no overlap in labelling by multiplex immunohistochemistry. All antibodies were α-Syn-specific (Supplementary Fig. 1) and exhibited no β/γ-synuclein cross-reactivity (Supplementary Fig. 2; only A15110D data shown). Potential differential antibody binding affinities were controlled for by conducting colocalisation validations with serial antibody titres (Supplementary Fig. 3A). The specificity of the N-terminus (34–45) antibody for α-Syn was further validated by immunolabelling with the other N-terminus antibody (MABN389; 44–57), which detects the epitope sequence immediately adjacent to that detected by 849102 (Supplementary Fig. 3B). Both N-terminus α-Syn antibodies exhibited comparable immunolabelling profiles. Due to supply and availability, we used 849102 for all subsequent investigations.

**Table 1:** Case information for human brain tissue used in this study.

Case	Neuropathological Diagnosis	Age	Sex	PMD (hrs)	Cause of death	Duration with disease (years)
PD53	PD/CLBD	79	F	25	Renal failure	9
PD54	PD	78	M	6	Aspiration pneumonia	19
PD56	PD/CLBD (neocortical/diffuse)	74	M	10.5	End stage Lewy body disease	12
PD57	PD/CLBD (neocortical/diffuse)	80	F	14	Bronchopneumonia	−
PD60	PD/CLBD (neocortical/diffuse)	80	M	18	Urosepsis	26
PD65	PD/CLBD (neocortical/diffuse)	67	M	2.25	End stage Parkinson’s disease	9
PD66	PD/CLBD (neocortical/diffuse)	73	M	17.5	Aspiration pneumonia	22
PD67	PD/CLBD (neocortical)	65	M	17	Pneumonia	12
PD77	PD/CLBD (limbic)	76	F	6.5	Abdominal carcinoma	23
PD79	PD/CLBD (neocortical)	77	M	6.5	End stage Lewy body disease	22
H245	Normal	63	M	20	Asphyxia	N/a
H250	Normal	93	F	19	Pneumonia	N/a

PMD, post-mortem delay; PD, Parkinson’s disease; CLBD, cortical Lewy body disease.

**Table 2:** Primary antibodies used for immunohistochemistry.

Antibody	Epitope	Species	Isotype	Manufacturer	Immunogenic Labelling	Catalogue No.	Dilution
α-synuclein A15110D	34–45	Mouse	Monoclonal IgG1	BioLegend	N-terminus	849102^15^	1:1000
α-synuclein 5G4	44–57	Mouse	Monoclonal IgG1	Merck	N-terminus	MABN389^31,32,71^	1:2000
α-synuclein	61–95	Chicken	Polyclonal	Abcam	NAC	Ab190376	1:1500
α-synuclein A15115A	80–96	Mouse	Monoclonal IgG1	BioLegend	NAC	848302^15^	1:1000
α-synuclein Clone 42	91–99	Mouse	Monoclonal IgG1	BD Biosciences	NAC/C-terminus	610787^53,72,73^	1:500
α-synuclein 4B12	103–108	Mouse	Monoclonal IgG1	ThermoFisher	C-terminus	MA1–90346	1:1000
α-synuclein	116–131	Sheep	Polyclonal	Abcam	C-terminus	Ab6162	1:1000
α-synuclein MJFR1	118–123	Rabbit	Monoclonal IgG	Abcam	C-terminus	Ab138501^74^	1:1000
α-synuclein 4D6	124–134	Mouse	Monoclonal IgG1	Abcam	C-terminus	Ab1903	1:1000
α-synuclein (pS129)	pS129	Mouse	Monoclonal IgG2a	Abcam	Phosphorylated serine 129	Ab184674^48,75^	1:4000
α-synuclein (pS129)	pS129	Rabbit	Monoclonal IgG	Abcam	Phosphorylated serine 129	Ab51253^48,71,74,75^	1:4000
β-synuclein	108–125	Rabbit	Polyclonal	Abcam	β-synuclein	Ab6165	1:500
γ-synuclein	**–**	Mouse	Monoclonal IgG2c	DSHB	Recombinant FL	CPTC- SNCG-1	1:200
NeuN	**–**	Guinea Pig	Polyclonal	Millipore	Neuronal nuclei marker	ABN90^76^	1:1000
MAP2	**–**	Chicken	Polyclonal IgY	Abcam	Neuronal cell marker	Ab5392	1:500
IBA1	**–**	Chicken	Polyclonal IgY	Synaptic Systems	Microglial/ macrophage cell marker	234006	1:1000
TMEM119	**–**	Rabbit	Polyclonal	Abcam	Microglial cell marker	Ab185333	1:500
GFAP	**–**	Chicken	Polyclonal IgY	Abcam	Astrocytic cell marker	Ab4674	1:3000
CNPase	**–**	Mouse	Monoclonal IgG2b	Novus Biologicals	Oligodendrocyte cell marker	CL2887	1:500
LAMP1	**–**	Mouse	Monoclonal IgG2a	Santa Cruz	Lysosomal marker	sc-17768	1:100
LAMP1	**–**	Mouse	Monoclonal IgG1	DSHB	Lysosomal marker	H4A3	1:100
LAMP2	**–**	Mouse	Monoclonal IgG1	DSHB	Lysosomal marker	H4A3	1:100
ALDH1L1	**–**	Mouse	Monoclonal IgG2b	ThermoFisher Scientific	Astrocyte cell/processes	UM500039CF	1:500

NAC, non-amyloid component; pS129, phosphorylated serine 129; DSHB, Developmental Studies Hybridoma Bank. Antigen retrieval was kept consistent for all antibodies (detailed in the methods sections).

**Fig. 2:**
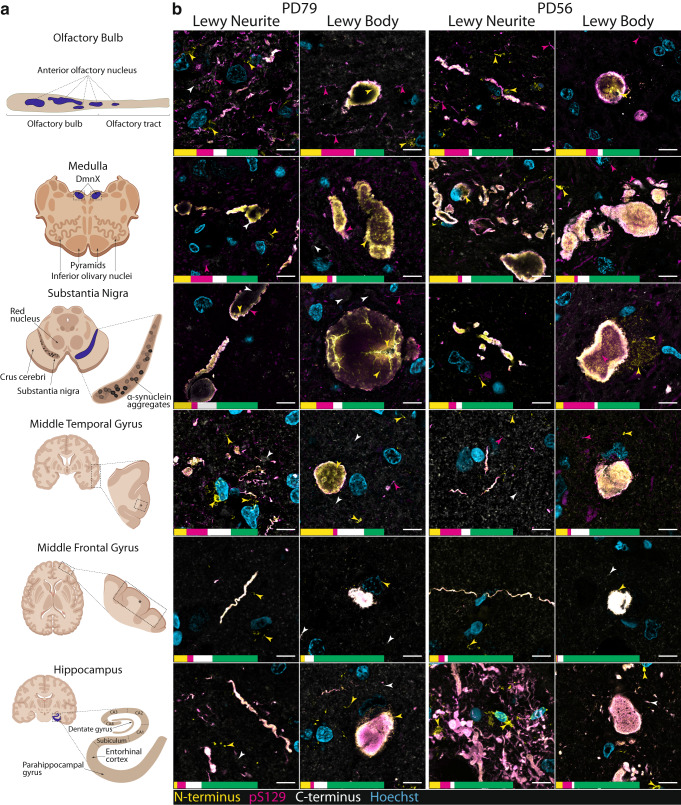
Representative confocal immunofluorescence images comparing α-Syn aggregate morphologies and epitope-specific α-Syn antibody immunoreactivities in representative Lewy neurites and Lewy bodies across pathologically significant regions of two human brains with PD. **a** Schematic diagram depicting the anatomical locations of each aggregate displayed in Figs. 2b and 3. Created with BioRender.com. **b** Yellow arrowheads indicate epitope-specific N-terminus immunoreactivity (AA 34–45), magenta arrowheads indicate epitope-specific pS129 immunoreactivity, and white arrowheads indicate epitope-specific C-terminus immunoreactivity (AA 118–123). Epitope-specific immunoreactivities and net overlapping immunoreactivities are depicted by the scaled percentage quantification bar. Quantifications recorded as percentage of total α-Syn immunolabelling area (yellow, epitope-specific N-terminus; magenta, epitope-specific pS129; white, epitope-specific C-terminus; green, overlapping immunolabelling). AA, amino acids; DmnX, dorsal motor nucleus of vagus nerve. Scale bar, 10 μm.

**Fig. 3:**
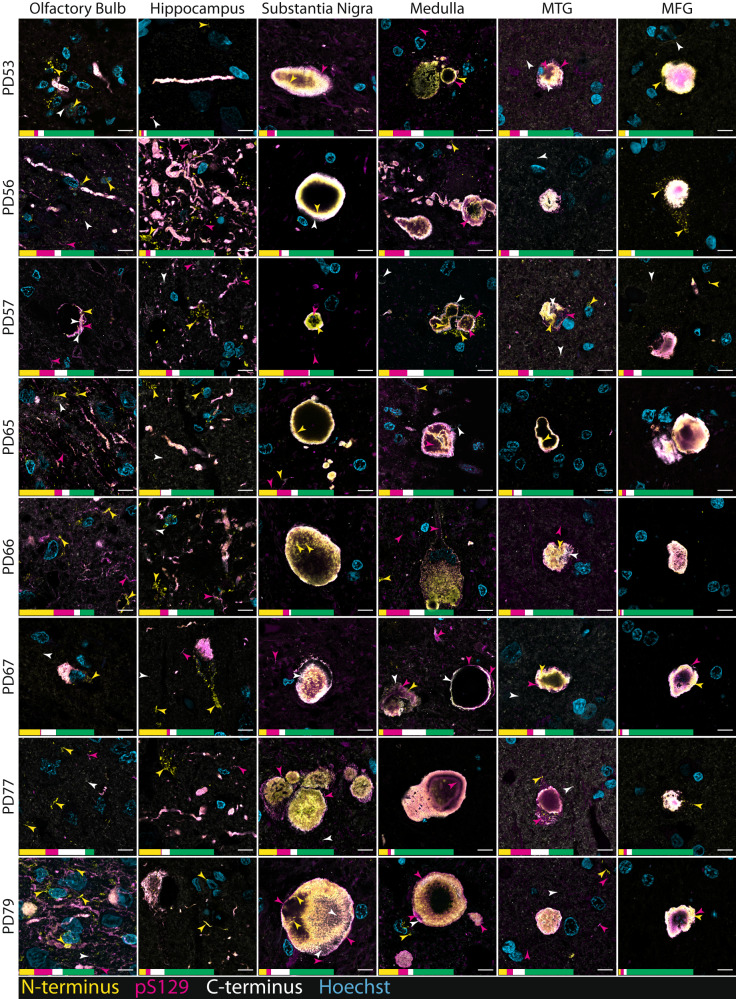
Representative confocal immunofluorescence images comparing α-Syn aggregate morphologies and epitope-specific α-Syn antibody immunoreactivities between pathologically significant regions of human brains with PD. Yellow arrowheads indicate epitope-specific N-terminus immunoreactivity (AA 34–45), magenta arrowheads indicate epitope-specific pS129 immunoreactivity, and white arrowheads indicate epitope-specific C-terminus immunoreactivity (AA 118–123). Epitope-specific immunoreactivities and net overlapping immunoreactivities are depicted by the scaled percentage quantification bar. Quantifications recorded as percentage of total α-Syn immunolabelling area (yellow, epitope-specific N-terminus; magenta, epitope-specific pS129; white, epitope-specific C-terminus; green, overlapping immunolabelling). All region-specific confocal images were acquired from the same relative anatomical locations (Fig. 2a). MTG, middle temporal gyrus; MFG, middle frontal gyrus; AA, amino acids. Scale bar, 10 μm.

Next, we used this validated α-Syn antibody panel to identify and quantify epitope-specific immunolabelling signatures in the human brain. For clarity, unique epitope-specific immunolabelling refers to pathological α-Syn that was exclusively detected by a single α-Syn antibody. Tissue sections were chosen from the olfactory bulb, medulla, substantia nigra, hippocampus, entorhinal cortex, middle temporal gyrus (MTG), and middle frontal gyrus (MFG) of ten PD cases (Fig. 2a). Lewy pathology was observed in all cases/regions and *unique* epitope-specific immunolabelling signatures – herein referred to as epitope-specific – were quantified for the N-terminus, pS129, and C-terminus antibodies in representative aggregates (Figs. 2b, 3). Distinct epitope-specific immunolabelling for each antibody was observed in both LB and LN α-Syn aggregate morphologies (Fig. 2b) across all studied brain regions (Fig. 3).

All epitope-specific α-Syn antibodies reliably identified LBs in all studied brain regions. Within individual LBs, distinct epitope-specific immunoreactivity profiles were observed. The pS129 and C-terminus antibodies tended to exhibit the greatest immunoreactivity at the periphery of individual LBs, reminiscent of the classical LB halo. pS129 and C-terminus immunoreactivity in the central region of LBs, whilst substantially less than at the periphery, was highly variable between aggregates. In contrast, the N-terminus antibody exhibited substantially more uniform immunoreactivity across LBs with diffuse immunolabelling typical within the central regions. These observations largely align with previously published data^25^.

In contrast to LB aggregate morphologies, LNs more frequently exhibited differential epitope-specific immunolabelling, highlighting the need for multiplex immunolabelling. To this end, we consistently observed two distinct populations of LNs across brain regions: (1) aggregates that were exclusively detected by the N-terminus antibody (Figs. 2b, 3; yellow arrowheads) and (2) aggregates that were exclusively labelled with the pS129 antibody (Figs. 2b, 3; magenta arrowheads). In contrast to these distinct LN populations, epitope-specific C-terminus immunoreactivity was largely restricted to the labelling of non-pathological (likely monomeric) α-Syn that did not exhibit any discernible aggregate morphology. This non-pathological α-Syn was defined based on its presence and appearance in neurologically normal cases, which is demonstrated in Supplementary Fig. 4.

To obtain a more representative insight into region/case-specific differential epitope-specific α-Syn immunoreactivities, a second round of quantifications was conducted on whole-tissue sections (Supplementary Fig. 5). To ensure that these quantifications were specific to pathological α-Syn, only defined α-Syn aggregates with threshold intensities above that of non-pathological monomeric α-Syn and non-specific background signatures were quantified. This was achieved by thresholding to ensure the exclusion of any non-pathological monomeric α-Syn that was also observed in neurologically normal cases. Across all regions, the mean epitope-specific pS129 and C-terminus immunoreactivity was 15 ± 11% and 10 ± 6%, respectively. Meanwhile the net overlapping immunoreactivity across all antibodies only accounted for 44 ± 20% of the total α-Syn immunolabelling area. Supplementary Fig. 5 shows the epitope-specific immunolabelling proportion of the N-terminus α-Syn antibody (percentage normalised to the total α-Syn aggregate immunolabelling area) and α-Syn pathology load (total combined aggregate immunolabelling area normalised to the total area of region quantified) for each case and region. The mean epitope-specific N-terminus immunolabelling was 38 ± 9% in olfactory bulb, 21 ± 10% in the medulla, 27 ± 12% in the substantia nigra, 23 ± 7% in the hippocampus (CA2), 40 ± 14% in the entorhinal cortex, and 34 ± 20% in both the MTG and MFG (Supplementary Table 1). A bar plot displaying the proportion of total α-Syn that exhibits exclusive N-terminus epitope immunoreactivity for each case and region is presented in Supplementary Fig. 6.

### Morphological distribution of α-synuclein pathology throughout the brain

**Fig. 4:**
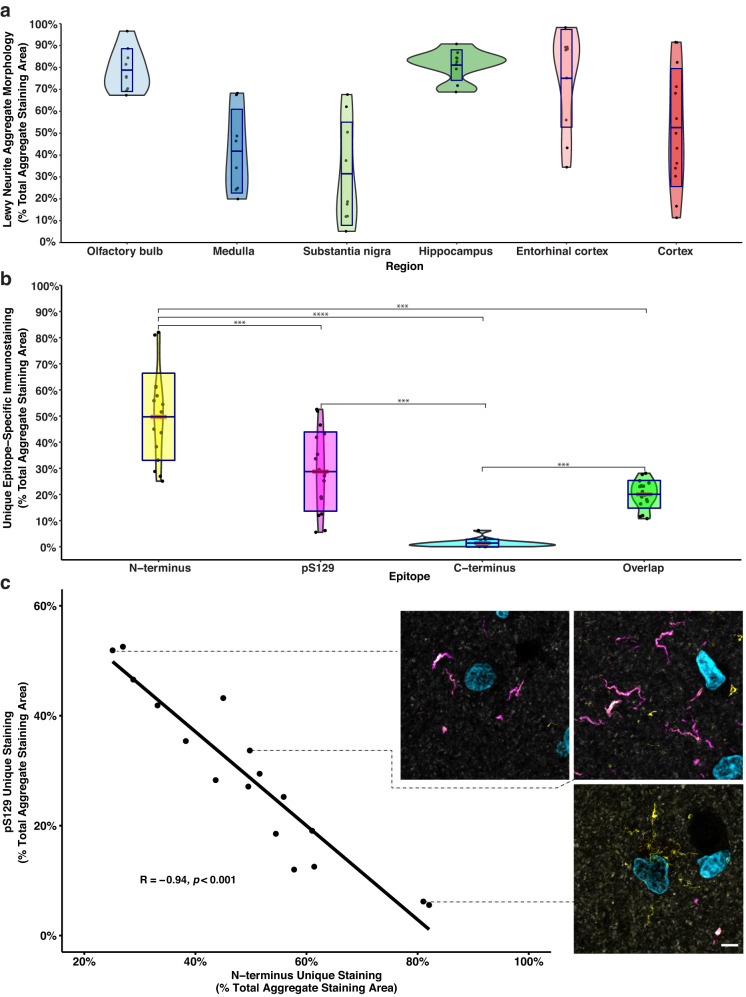
Morphological distribution of Lewy pathology across studied regions and quantification of epitope-specific α-Syn immunolabelling in a tissue microarray of the PD MTG. **a** Violin plot depicting the relative abundance of Lewy neurite aggregate morphologies across pathologically significant regions of the human brain with PD. Crossbar = mean; boxplot = error bars (±SD); whiskers = range. **b** Violin plot depicting the mean and relative distribution of epitope-specific immunoreactivity across PD cases in a tissue microarray of the human MTG. Mean epitope-specific N-terminus immunolabelling was significantly higher than for the C-terminus epitope across cases (*p* < 0.0001, one-way ANOVA followed by Tukey’s multiple comparison test). Mean epitope-specific pS129 immunolabelling was significantly higher than for the C-terminus epitope across cases (*p* < 0.001, one-way ANOVA followed by Tukey’s multiple comparison test). ****p < 0.0001, ***p < 0.001. Crossbar = mean; boxplot = error bars ( ± SD); whiskers = range. **c** N-terminus and pS129 immunoreactivity exhibited a strong negative correlation (*r* = -0.94, *p* < 0.001, Pearson correlation coefficient). Representative confocal images from cases with high, medium, and low epitope-specific N-terminus immunolabelling (yellow, N-terminus; magenta, pS129; cyan, C-terminus). Scale bar, 5 μm.

Several neuropathological staging frameworks that characterise the maturation and interregional progression of α-Syn pathology in LB disorders are well-documented and are widely accepted within literature^36–40^. By and large, however, these seminal works rely on histological stains or single-label immunohistochemical approaches, which do not capture the full cohort of α-Syn variants present. In light of this, we used multiplex fluorescence immunohistochemistry with quantitative analysis to measure the distribution of α-Syn aggregates in different regions of the human brain affected by LB disease (Fig. 4a).

In the olfactory bulb, pathology is localised within the anterior olfactory nucleus. Small-medium-sized thread-like LNs predominated, however, the occasional LB was often interspersed (Figs. 2b, 3, Supplementary Fig. 7). Across cases, LN aggregate morphologies accounted for 78.83% of the total pathology in the olfactory bulb (Fig. 4a; LBs, 21.17%). In line with previous findings, the amount of aggregated α-Syn outside of the anterior olfactory nucleus was minimal in all cases^41^. In the hippocampus, a dense web of LNs was consistently observed spanning the CA2 subregion of the hippocampus proper (Fig. 2b, PD56 Hippocampus, Supplementary Fig. 7), reminiscent of those previously reported by Braak et al.^36^. This band-like LN web, whilst most prominent within the CA2 subregion, also extended to the adjacent CA1 and CA3 subregions in most cases. All major LN morphologies were observed within this grouping, including large club-shaped, long thread-like, and short tadpole-like morphologies; several small LBs were interspersed throughout (Supplementary Fig. 7). Collectively, LN aggregate morphologies accounted for 81.11% of the total α-Syn immunolabelling area (Fig. 4a) within the hippocampus proper (LBs, 18.89%). Within the adjacent cortical regions, namely, the entorhinal cortex and the parahippocampal gyrus, small LBs, LNs and Lewy dots were consistently observed. In addition to these conventional aggregate morphologies, glial aggregates exhibiting exclusive N-terminus immunoreactivity were also prominent within the entorhinal cortex and parahippocampal gyrus (Supplementary Fig. 7). Within the entorhinal cortex, LN morphologies accounted for 75.05% of the total α-Syn immunolabelling area (Fig. 4a; LBs, 24.95%).

Within the medulla, the morphological distribution of α-Syn aggregates was relatively comparable across cases, with LNs accounting for 41.76% of the total α-Syn immunolabelling area (Fig. 4a) and LBs accounting for 58.24%. Aggregates were almost exclusively localised to the dorsal motor nucleus of the vagus nerve; however, in some cases (PD56, PD65, PD66, PD77) aggregates were also occasionally observed in immediately adjacent regions. Specific LN and LB morphologies varied extensively across aggregates (Supplementary Fig. 7). LNs ranged in size and morphology from short thread-like aggregates to large club-shaped aggregates. LBs, in contrast, were characteristically smaller in size, ranging from ~5–15 μm. Several large extracellular LBs, however, were also observed, ranging from ~13–43 μm. Interestingly, the vast majority of LBs did not exhibit an intraneuronal disposition but instead appeared to be extracellular. Dense mixed-morphology aggregate populations were also commonly observed, with several LNs and LBs clustering in close proximity to one another.

The diverse range of LN morphologies previously described were also present within the substantia nigra, however, the larger club-shaped morphology predominated (Fig. 2b). In contrast to other regions, most α-Syn immunopositive aggregates in the substantia nigra exhibited a distinct LB morphology, accounting for 68.55% of the total α-Syn immunolabelling area (LNs, 31.45%; Fig. 4a). In addition to classical intracellular LBs, substantially larger extracellular LBs, that were completely devoid of surrounding neuromelanin, were common (Figs. 2b, 3, Supplementary Fig. 7). In several instances, the diameters of these extracellular LBs measured in excess of 50 μm (Figs. 2b, 3, Supplementary Fig. 7). Moreover, within nigral sections, individual LBs were commonly observed to be in direct contact with one another, forming more complex multi-aggregate structures (Fig. 3, PD77). Finally, the distribution of aggregate morphologies within cortical regions (MTG and MFG) was highly variable across cases. Across cortical regions, LNs accounted for 52.54% of the total α-Syn immunolabelling area (Fig. 4a), with LBs accounting for 47.46%. Small-medium-sized intracellular LBs (7–21 μm) and small-medium length thread-like LNs (~3–25 μm) predominated (Fig. 2b) and largely localised within the grey matter.

### Differential epitope-specific α-synuclein immunolabelling within the middle temporal gyrus

To investigate whether epitope-specific immunolabelling was consistently observed in a larger group of cases, we immunolabelled tissue microarray (TMA) sections that contained 2 mm cores from the MTG of PD (*n* = 23) and neurologically normal (*n* = 24) cases with our selected antibody panel. The MTG region was selected due to its relatively homogeneous distribution of α-Syn pathology. The α-Syn pathology load within the neurologically normal patient cohort was minimal. Quantitative image analysis revealed extensive differential epitope-specific immunoreactivities in all PD cases (Fig. 4b, Supplementary Fig. 8). The mean epitope-specific immunolabelling (percentage normalised to the total α-Syn aggregate immunolabelling area) in the PD cohort was 50 ± 16% and 29 ± 15% for the N-terminus and pS129 epitopes, respectively. Epitope-specific N-terminus and pS129 immunolabelling exhibited a strong negative correlation (Fig. 4c; *r* = -0.94, *p* < 0.001, Pearson correlation coefficient). Contrastingly, mean epitope-specific C-terminus immunoreactivities were significantly lower compared to N-terminus and pS129 (*p* < 0.0001, one-way ANOVA followed by Tukey’s multiple comparison test), only accounting for 1.4 ± 0.6% of the total α-Syn aggregate immunolabelling (Fig. 4b). Although epitope-specific α-Syn immunoreactivities were observed across all PD cases, the amount was highly variable across the cohort (Supplementary Fig. 8). Importantly, net overlapping α-Syn immunolabelling (calculated by subtracting the epitope-specific immunolabelling area of each antibody from the total α-Syn immunolabelling area) only accounted for 20 ± 5% of the total α-Syn immunolabelling area across PD cases (Fig. 4b), indicating the majority of the total α-Syn labelling is epitope-specific labelling.

### Epitope-specific immunolabelling reveals distinct α-synuclein aggregate morphologies

**Fig. 5:**
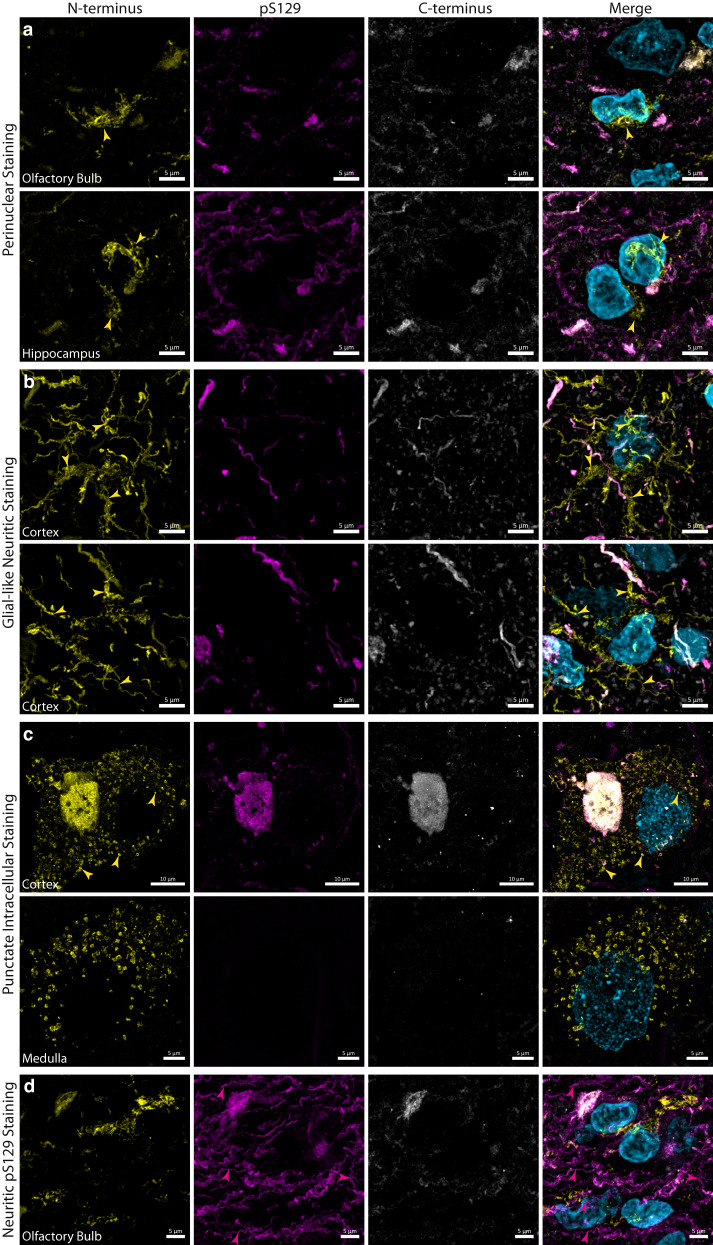
Representative confocal immunofluorescence images demonstrating morphologically distinct α-Syn aggregates that exhibit exclusive N-terminus α-Syn immunoreactivity. **a** Perinuclear α-Syn aggregates that exhibited exclusive N-terminus immunoreactivity (yellow arrowheads) were observed across all interrogated brain regions. **b** Glial neuritic immunolabelling was observed in cortical regions of the medial temporal lobe, particularly within the entorhinal cortex and surrounding limbic regions of the parahippocampal gyrus. **c** Distinct populations of punctate intracellular α-Syn that exhibited exclusive N-terminus immunoreactivity (yellow arrowheads) were also observed across all interrogated brain regions. **d** Distinct populations of aggregated α-Syn that exhibited exclusive pS129 immunoreactivity (magenta arrowheads) were observed across all regions but were most abundant in the olfactory bulb and hippocampus. Scale bar, 10 μm.

Immunolabelling of the N-terminus consistently detected three morphologically distinct α-Syn aggregate populations that were not detected by pS129 and C-terminus immunolabelling. The first population exhibited a thin LN thread-like morphology that was largely perinuclear in localisation (Fig. 5a). Perinuclear aggregates were abundant across all anatomical regions studied. Secondly, distinct glial aggregates were observed in cortical regions of the temporal lobe, particularly within the entorhinal cortex and surrounding parahippocampal gyrus (Fig. 5b). The third aggregate population exhibited a distinct punctate intracellular morphology and was localised to neuronal cell bodies and processes (Fig. 5c). This punctate intracellular aggregate morphology was consistently seen within the medulla, substantia nigra, MTG, MFG, the CA2 subregion of the hippocampus proper, and the surrounding parahippocampal gyrus. Finally, distinct populations of neuritic α-Syn aggregates exhibiting exclusive pS129 immunolabelling were observed within the anterior olfactory nucleus of the olfactory bulb, the hippocampus and entorhinal cortex, as well as the MTG (Figs. 4, 5d).

**Fig. 6:**
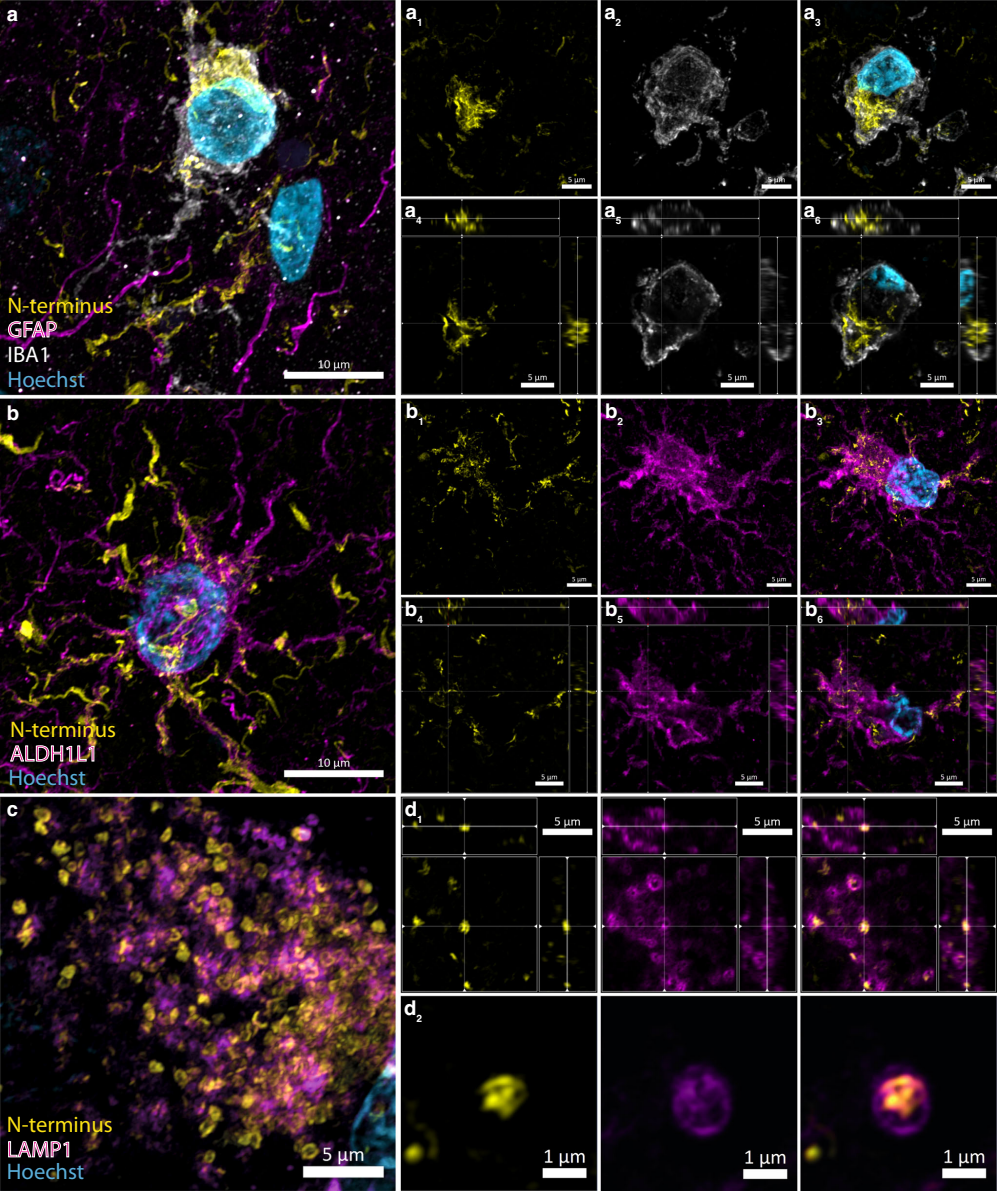
Representative confocal immunofluorescence images validating the glial and lysosomal localisation of epitope-specific N-terminus α-Syn immunoreactivity. **a** Microglial (IBA1; cyan) and (**b**) astrocytic (GFAP; magenta) localisation of N-terminus α-Syn (yellow). **a**_**1–6**_, **b**_**1–6**_ Representative confocal orthogonal slices validating the intracellular localisation of N-terminus α-Syn within **a**_**1–6**_ microglia, and **b**_**1–6**_ astrocytes. **c,** **d**_**1**_, **d**_**2**_ Representative confocal images and orthogonal slices validating the lysosomal localisation (LAMP1; magenta) of N-terminus α-Syn (yellow).

We used the macrophage/microglial markers, IBA1 and TMEM119, and astrocytic markers, GFAP and ALDH1L1 (Table 2), to further investigate the cellular localisation of the glial aggregates. We found that these glial aggregates largely localised within the cell bodies and processes of microglia (Fig. 6a, 6a_1–6_) and astrocytes (Fig. 6b, b_1–6_). In some instances, however, the glial aggregate processes did not entirely colocalise with glial markers and, instead, were in close proximity to microglial and astrocytic processes (Fig. 6a_3_, b_3_). Furthermore, colocalisation validations with a lysosomal marker (LAMP1) revealed that the punctate intracellular aggregates largely localised within neuronal lysosomes (Fig. 6c, c_1–6_). The two antibodies that detect the late N-terminus (MABN389; 44–57) and NAC (848302; 80–96) domains also labelled these glial and lysosomal aggregate morphologies (Supplementary Fig. 3, Supplementary Fig. 9). No N-terminus immunoreactivity was observed in neurologically normal cases, thereby mitigating lysosomal or glial cross-reactivity of the N-terminus antibody as a confounding factor.

### N-terminus immunoreactive conformers are susceptible to proteolytic digestion

**Fig. 7:**
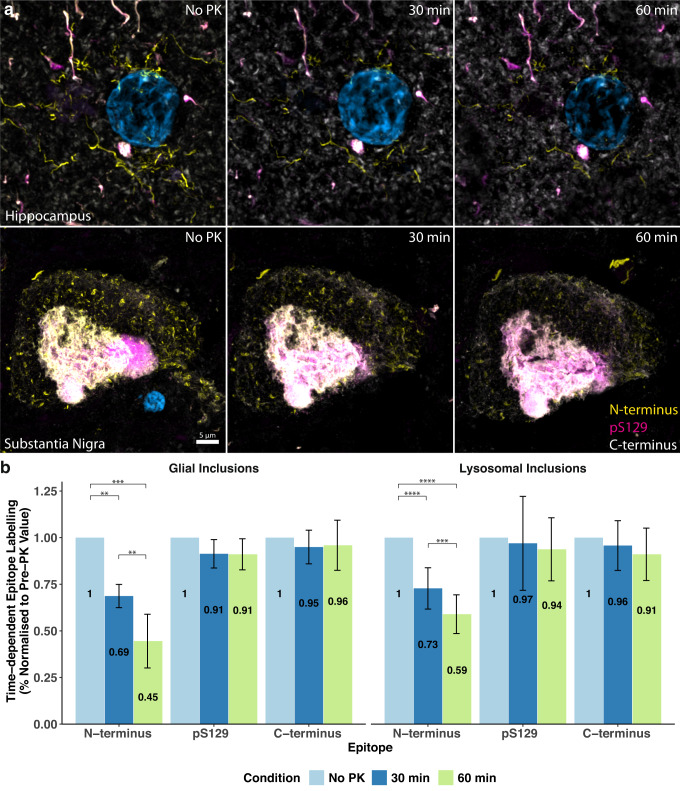
Epitope-specific quantifications demonstrating the time-dependent proteolysis of the N-terminus (34–45) α-Syn epitope and relative preservation of pS129 and C-terminus (118–123) epitopes in single aggregates following proteinase K treatment. **a** Representative confocal orthogonal projections (acquired with identical imaging parameters and 3D Cartesian coordinates) of N-terminus immunoreactive glial (top) and lysosomal (bottom) aggregate morphologies in the hippocampus and substantia nigra following time-dependent proteinase K digestion. **b** Quantification of epitope-specific immunolabelling in single representative aggregates acquired from PD cases (*n* = 8). All quantifications are normalised to the pre-treatment (No PK) immunolabelling values. The N-terminus epitope progressively degraded over time, whereas the pS129 and C-terminus (118–123) epitopes remained relatively intact. PK, proteinase K. Data is presented as mean ± SD. ****p < 0.0001, ***p < 0.001, **p < 0.01; repeated-measures one-way ANOVA followed by Tukey’s multiple comparison test. Scale bar, 5 μm.

To characterise the aggregation state of the glial and lysosomal α-Syn aggregates, we conducted a time-dependent proteinase K digestion of hippocampal (*n* = 8) and substantia nigra (*n* = 8) tissue sections. The degradation of both glial and lysosomal aggregates in the same representative α-Syn aggregates, following both 30 min and 60 min of proteinase K digestion, was quantified (Fig. 7). Interestingly, there was no α-Syn degradation following 15 min of proteinase K digestion in either the hippocampus or the substantia nigra. In fact, this initial 15 min proteinase K digestion timepoint actually unmasked each α-Syn epitope to a greater degree than their pre-treatment detection levels, highlighting the potential benefits of incorporating proteinase K treatment as part of standard aggregate-based epitope retrieval methodologies. Degradation of the N-terminus was evident following 30 min and 60 min of proteinase K digestion (Fig. 7). At 30 min, the glial aggregates were digested to 68.7 ± 2.4% (*p* = 0.0001) of their pre-treatment immunolabelling quantity; this was reduced to 44.5 ± 5.4% (*p* = 0.0002) at 60 min (Fig. 7b, Hippocampus). Following 30 min of proteolytic digestion, the lysosomal aggregates were digested to 72.8 ± 3.5% (*p* < 0.0001, repeated-measures one-way ANOVA followed by Tukey’s multiple comparison test) of their pre-treatment quantity, which was reduced to 58.9 ± 3.3% (*p* < 0.0001, repeated-measures one-way ANOVA followed by Tukey’s multiple comparison test) by 60 min (Fig. 7b; Substantia Nigra). The degradation of the N-terminus epitope from 30–60 min was statistically significant in both aggregate populations (Glial Inclusions, *p* = 0.005; Lysosomal Inclusions, *p* = 0.0008, repeated-measures ANOVA followed by Tukey’s multiple comparison test). Importantly, whilst there was clear degradation of the N-terminus epitope (34–45) in representative aggregates, the pS129 and C-terminal (118–123) epitopes remained largely intact and detectable relative to their pre-treatment levels (Fig. 7b). These findings were further corroborated when quantified in whole-tissue section acquisitions (Supplementary Fig. 10).

### Maturation of extracellular Lewy bodies

**Fig. 8:**
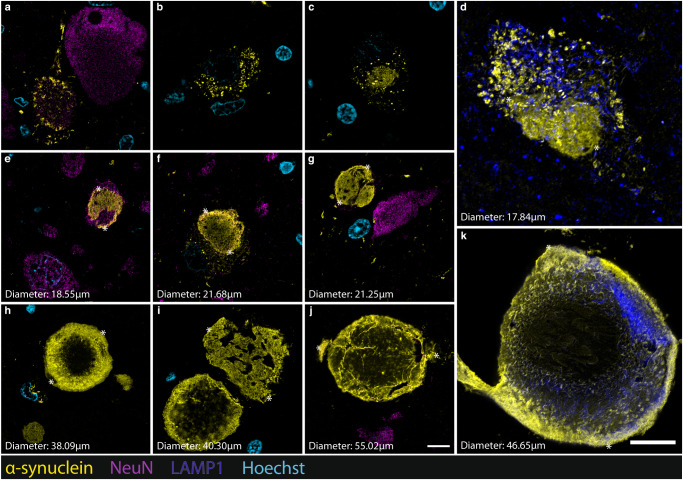
Representative confocal images depicting the maturation of Lewy body pathology (N-terminus α-Syn; 849102) in the human brain with PD. **a**, **b** Granular and punctate α-Syn aggregates begin to accumulate in the neuronal cytoplasm but do not yet exhibit a structured morphology. **c** Irregularly shaped aggregates begin to condense and expand within the cytoplasm. **d**, **e** Aggregates continue to expand within the confines of the intracellular space and gradually become more structurally well-defined and uniformly labelled. **f** Classical Lewy body morphology becomes structurally well-defined, characterised by a peripherally condensed halo. **g**, **h** Aggregates eventually outgrow the confines of the intracellular space and rupture the cellular membrane, allowing growth to continue within the extracellular space. **i** Distinct Lewy bodies can interact with one another and (**j**, **k**) merge to form compound extracellular aggregate structures. Asterisks define the distance measured in each image. Scale bar, 10 μm.

In characterising the distribution of α-Syn aggregate morphologies across brain regions, we identified significant populations of large extracellular LBs within the medulla and substantia nigra, and to a lesser extent, the hippocampus and cortical regions. Whilst large extracellular LB morphologies have previously been described^25,40,42–44^, these existing pathological staging frameworks of LB maturation largely lack characterisation of how these extracellular LBs continue to develop beyond the intracellular stage. Here, we propose a hypothetical staging framework that details how LBs might continue to mature beyond the confines of the intracellular space (Fig. 8). Across our interrogated regions, we observed N-terminus epitope labelling resembling that which has previously been described by existing frameworks of LB maturation (Fig. 8)^25,40,44^. Specifically, we observed fine, granular labelling that is associated with the initial phase of LB aggregation (Fig. 8; a-c). As previously detailed, we consistently observed a greater proportion of this granular α-Syn that was exclusively N-terminus immunoreactive, compared to that also labelled with pS129 and C-terminus antibodies (Figs. 2, 3 and 4c). N-terminus immunolabelling consistently detected LB precursor aggregates (Fig. 8; d, e), classically termed *pale bodies*, within the confines of the intracellular space, and clearly labelled the distinct central core and dense α-Syn-immunopositive peripheral halo of mature LBs (Fig. 8; f). Finally, we observed N-terminus labelling of LBs that do not appear to be intracellular, being considerably larger than and not associated with surrounding NeuN-labelled cell bodies or Hoechst-stained nuclei (Fig. 8; g, h). Within regions of high pathology, namely the medulla and substantia nigra, we consistently observed instances in which these large, extracellular ‘ghost’ LBs were in close proximity with one another in the extracellular space (Fig. 8; I). The large extracellular LBs ranged from 40–55 µm in diameter (Fig. 8; j, k, Supplementary Fig. 7).

## Discussion

The precise and complete capture of all α-Syn variants in the human brain is imperative to ensuring that accurate and meaningful conclusions can be drawn from immunohistochemical interrogations of human α-synucleinopathies. α-Syn is an inherently complex protein. In addition to undergoing extensive post-translational modification, including phosphorylation, ubiquitination, nitration and truncation, α-Syn also exhibits extensive microenvironment-dependent conformational variability within the human brain. Given the extent to which researchers rely on antibodies to inform their understanding of α-Syn and its role in human α-synucleinopathies, it is critical that the specific epitopes these antibodies detect are considered with reference to the protein’s complex and variable structure and various PTMs. In the present study, we interrogated the bulk of α-Syn with antibodies that detect all structural domains and revealed novel α-Syn aggregate morphologies that are only detected with N-terminus (34–57) and NAC (80–96) epitope immunolabelling. We identified and characterised distinct epitope-specific immunoreactivities in all seven pathologically significant regions interrogated. Whilst the extent of differential immunolabelling varied between regions, the N-terminus consistently exhibited the greatest proportion of unique epitope-specific immunoreactivity across cases and regions.

Secondary to the N-terminus, the pS129 PTM also exhibited a moderate degree of unique epitope-specific immunolabelling, particularly within the olfactory bulb, medulla, substantia nigra, and across the MTG TMA cores. Given the high pathological abundance of C-terminally-truncated α-Syn, this finding was somewhat surprising, especially when considering the high pS129 epitope-specific immunolabelling and strong negative correlation between the N-terminus and pS129 epitope immunoreactivities across the MTG TMA cores. These exclusively pS129 immunoreactive aggregates could potentially represent N-terminally-truncated fragments that still have the C-terminus intact. Alternatively, they could be explained by PTMs that affect α-Syn at the N-terminal Y39 residue. C-Abl protein tyrosine kinase phosphorylates Y39, which protects α-Syn from degradation, at least in cortical neurons^45,46^. pY39 attracts the lysine residues of the N-terminus, which induces a conformational fold, resulting in the formation of a hydrophilic N-terminus fibril core with pY39 at the centre of the α-Syn polymorph^46^. This profound conformational change could potentially mask residues 34–45, in certain α-Syn aggregate morphologies, thereby preventing the detection of these protofilaments by the N-terminus antibody, whilst preserving their pS129 immunoreactivity. Y39 can also be selectively nitrated (nY39), which might further impede the ability of the N-terminus antibody to bind to its target epitope sequence^47^. nY39 abundance is highest in regions earliest affected by α-Syn pathology, which could partially explain the higher levels of epitope-specific pS129 immunoreactivity observed in the olfactory bulb and medulla^47^. Further investigations with PTM-specific antibodies targeting the Y39 residue of α-Syn would help to validate this hypothesis.

In contrast to N-terminus and pS129 immunoreactivity, epitope-specific C-terminus immunoreactivity was largely restricted to endogenous, putatively non-pathological α-Syn. This observation was consistent across all C-terminus α-Syn antibodies investigated. One explanation is that these C-terminus antibodies are directed against epitope sequences that include at least one of Asp-119 or Asn-122, the two major truncation sites of α-Syn, or other prominent C-terminus truncation sites. Consequently, in the absence of a full epitope sequence to bind to, these C-terminus antibodies will fail to detect several pathologically significant C-terminally truncated variants of α-Syn and, instead, only detect full-length α-Syn, much of which is still present in its native unfolded form. Importantly, across all interrogated regions, the overlapping (non-epitope-specific) immunoreactivity across epitopes only accounted for 44 ± 20% of the total α-Syn immunolabelling area, and 20 ± 5% across all MTG cores in the TMA. Considering the toxicity of the N-terminus and C-terminally truncated α-Syn in the process of fibril formation, and the pathological pertinence of the pS129 PTM, this data highlights the necessity of utilising multiple epitope-specific antibodies to accurately capture the diversity of pathogenic α-Syn variants present in the human brain^2,17–24^.

The most intriguing finding of the present study was our identification of multiple morphologically distinct α-Syn populations that exhibit exclusive N-terminus immunoreactivity. This finding is of particular significance given that antibodies targeting the C-terminus of α-Syn, including pS129, are so heavily relied upon by research and pathology laboratories worldwide^8,12,48^. This single-domain approach risks the incomplete detection of the wider range α-Syn conformers present in the human brain. Furthermore, future α-Syn clearing treatments may rely on pS129 clearance and thus leave other epitopes in situ. Our findings suggest that routine detection of the N-terminus (namely residues 34–57) and NAC domains (residues 80–96) in research and diagnostics would improve data accuracy and interpretation.

Pathological α-Syn is generally considered to be cleared within lysosomes, via the Autophagy-Lysosome Pathway, with truncational α-Syn variants forming due to incomplete lysosomal degradation^26,49–51^. In addition, the non-neuronal uptake of both monomeric and aggregated forms of α-Syn is well established, with several publications reporting astrocytic and microglial immunoreactivity of N-terminus and NAC α-Syn epitopes^15,32,52–57^. Whilst the mechanisms underlying the astrocytic uptake of α-Syn remain to be fully elucidated, oligomeric α-Syn is rapidly internalised by astrocytes in vitro^58,59^. Upon ingestion, this internalised α-Syn is targeted for degradation via the lysosomal pathway^58–60^. This astrocytic accumulation of internalised α-Syn, however, impairs astrocytic phagosomal-lysosomal machinery, leading to the incomplete degradation of α-Syn^58,59^. This ultimately results in the intracellular accumulation of oligomeric α-Syn, which induces subsequent mitochondrial defects^58,59^. In contrast to astrocytes, Toll-like receptor 4 (TLR4) mediates microglial uptake and clearance of α-Syn^56,61^. Choi et al. reported that microglia protect neurons by clearing extracellular α-Syn through selective autophagy, termed “synucleinphagy”^61^. Through this process, engulfed α-Syn is sequestered by microglia into autophagosomes for lysosomal degradation, via TLR4-mediated signalling. Despite being the main cell type responsible for the clearance of extracellular protein aggregates, recent evidence suggests that microglial degradation of fibrillar α-Syn may also be compromised under high α-Syn loads^62^.

The significant proportion of early-stage granular α-Syn aggregates that we observed to be exclusively N-terminus immunoreactive suggests that C-terminally truncated fragments of α-Syn could play an important role in the early pathogenesis of Parkinson’s disease. Considering the recent evidence implicating the N-terminus of C-terminally truncated α-Syn in the acceleration of fibril formation and increased mitochondrial damage, future studies should look to elucidate the conformational properties and functional role of these N-terminus polymorphs^24^.

The susceptibility of the epitope-specific N-terminus immunoreactive aggregate morphologies to proteolytic digestion suggests they are predominantly constituent of soluble non-fibrillar forms of α-Syn and may represent a less stable oligomeric aggregation state. Several studies investigating the structure of α-Syn oligomers indicate that the N-terminus and NAC domain concentrate largely within the oligomeric core, whereas the C-terminus remains exposed^63–65^. This oligomeric conformational architecture inevitably leaves the C-terminus vulnerable to lysosomal degradation, whilst conformationally protecting epitopes within the N-terminus and NAC domains. Conformation-induced resistance of the N-terminus and NAC domains to degradation could explain the epitope-specific N-terminus (34–57) and NAC (80–96) immunoreactivity of glial aggregates, as well as the high degree of lysosome-aggregate colocalisation. In addition, the absence of detection of these epitope-specific α-Syn aggregate morphologies by the NAC (61–95) antibody could potentially be explained by conformational-induced masking of target epitopes within the early-mid NAC region (61–79). To this end, antibodies can only bind to epitopes that are exposed within the compact conformational arrangement of misfolded α-Syn. It is possible that aggregates constituent of full-length or truncated α-Syn variants could be conformationally arranged such that target epitopes within the NAC domain and C-terminus are obscured, whilst residues 34–57 of the N-terminus and 80–96 of the NAC domain remain exposed. Future investigations using α-Syn antibodies that detect additional epitope sequences across the N-terminus and NAC domain would help to further interrogate both these hypotheses.

Finally, whilst large extracellular LB morphologies have previously been described, existing pathological staging frameworks of LB maturation lack characterisation of how these extracellular LBs continue to develop beyond the intracellular stage^42,66^. We propose that mature LBs, like a sinkhole, continue to sequester α-Syn, as well as other soluble proteins and organelles within their vicinity. As they grow, LBs impart substantial damage to cellular machinery and gradually compromise the structural integrity of the surrounding cellular membrane, which ultimately ruptures to release the LB into the extracellular space. The resulting extracellular *ghost* LBs are analogous to the ghost neurofibrillary tangles that are observed in Alzheimer’s disease. Although image acquisitions only represent a snapshot in time, we propose that ghost LBs in close proximity engage in dynamic interactions with one another, which facilitate their ability to merge with one another to form super-sized extracellular aggregates. These findings prompt further validations of the nature of these potential interactions, which would benefit from interrogation with super-resolution techniques, such as cryogenic electron microscopy.

Taken together, the present study highlights the importance of employing a multiplex immunohistochemical approach to more accurately capture the diversity of α-Syn variants present within α-synucleinopathies. The susceptibility of α-Syn to post-translational and conformational modification underscores its structural complexity, with several of these structural variants driving the heterogeneity of α-synucleinopathic pathogenesis. Such complexity necessitates more comprehensive investigative toolsets that employ multiplex detection with a range of well-characterised epitope-specific and PTM-specific α-Syn antibodies to ensure optimal detection and consideration of these multifaceted structural variants.

## Methods

### Human brain tissue acquisition and processing

Human post-mortem brain tissue was obtained from the Neurological Foundation Human Brain Bank. The Brain Bank sits within the University of Auckland, and is regulated by an act of the New Zealand Parliament with oversight by the New Zealand Police. The studies were approved by the NZ Health and Disabilities Ethics Committee and by the University of Auckland Human Participants Ethics Committee (Ref: 011654). All brain tissue was donated with written informed consent from donors and their families prior to brain removal. All experiments were conducted in accordance with relevant guidelines and regulations. All cases used in this study were assessed by a neuropathologist. The neurologically normal cases (*n* = 24) had no clinical history of neurological abnormalities, and no other significant neuropathology was noted upon post-mortem examination. The mean age (±standard deviation) of normal cases was 69 ± 15 and ranged from 35–98 years (Table 1, Supplementary Table 2). The mean post-mortem delay of normal cases was 20 ± 9 h with a range of 4–48 h. All PD cases (*n* = 25) had a clinical history of PD, and pathological features were consistent with PD pathology as confirmed by a neuropathologist. Key neuropathological features were loss of pigment and pigmented cells in the substantia nigra and accumulation of LBs in the substantia nigra and other brain regions; many cases also had evidence of cortical LB disease. PD cases had a disease duration ranging from 1–26 years; the mean duration was 15 ± 7 years (Table 1, Supplementary Table 2). The mean age of PD cases was 78 ± 8 and ranged from 60–91 years; the mean post-mortem delay was 12 ± 7 h with a range of 2.25–25 h (Table 1, Supplementary Table 2).

Upon receipt of the brain, the right hemisphere of each brain was fixed by perfusion of 15% formaldehyde in 0.1 M phosphate buffer through the cerebral arteries and subsequently dissected into anatomically significant blocks as previously described^67,68^. A 5 mm-thick section was sampled from each block for paraffin embedding and the remaining tissue was snap-frozen using powdered dry ice and stored at -80°C. All olfactory bulbs were removed from the brain prior to perfusion to preserve olfactory tissue integrity. Olfactory bulbs were subsequently immersion fixed in 15% formaldehyde in 0.1 M phosphate buffer for 24 h at room temperature. Both the brain tissue blocks and olfactory bulbs were processed for paraffin embedding as previously described^69^. Paraffin blocks were sequentially sectioned using a rotary microtome (Leica Biosystems, RM2335) at a thickness of 7 μm. Olfactory bulb blocks were sectioned in the sagittal plane, medulla oblongata and substantia nigra blocks were sectioned in the horizontal plane, and hippocampal and cortical blocks were sectioned in the coronal plane. Sections were individually mounted onto Über plus charged microscope slides (IntstrumeC) using a 41°C-water bath (Leica Biosystems, H1210). Mounted sections were desiccated at room temperature for 72 h.

### Tissue microarray construction

A TMA was constructed using 2 mm paraffin-embedded formalin-fixed cores of PD (*n* = 23) and neurologically normal (*n* = 24) MTG grey matter, as described previously^70^. The MTG was selected due to its relatively homogeneous distribution of α-Syn pathology. Briefly, 2 mm tissue cores were extracted from both PD and neurologically normal donor blocks using an Advanced Tissue Arrayer (VTA-100, Veridiam). Donor tissue cores were then inserted into a blank recipient TMA paraffin block to form an array of cores. Following heat-induced adherence of the donor cores to the recipient block, the recipient block was sequentially sectioned at a thickness of 7 μm, as detailed above. Case information for all cases arrayed on the TMA is presented in Supplementary Table 2. PD and neurologically normal cases were matched for both age and post-mortem delay. Due to the inherent sampling limitations of TMAs, damaged cores, or cores with no pathology load (*n* = 5) were omitted from subsequent analysis.

### Immunohistochemistry

Mounted formalin-fixed paraffin-embedded tissue sections were heated on a 60 °C hot plate for 1 h to melt the embedding paraffin. Paraffin wax was removed by submerging tissue sections in two consecutive 100% xylene baths (2 × 30 min). Tissue sections were rehydrated by sequentially immersing slides in a series of ethanol baths (100% EtOH, 2 × 15 min; 95%, 80% and 75% EtOH, 1 × 10 min) followed by 3 × 5 min washes in distilled H_2_O. Heat-induced epitope retrieval was performed by heating slides in a Tris-EDTA (pH 9.0, 0.05% Tween 20) buffer (Abcam) in a pressure cooker (2100 Antigen Retriever, Aptum Biologics Ltd.) at 121°C for 20 min. Sections were cooled for 1.5 h and washed in distilled H_2_O (3 × 5 min). Secondary acid-induced epitope retrieval was performed by incubating sections for 4 min in 99% formic acid, after which sections were washed in distilled H_2_O (3 × 5 min). Hydrophobic wax barriers were drawn around tissue sections using an ImmEdge Hydrophobic Barrier PAP pen (Vector Laboratories) and sections were permeabilised for 15 min in 4°C PBS-T (PBS with 0.2% Triton™ X-100; Sigma-Aldrich, T9284). Tissue sections were washed (3 × 5 min in PBS) and incubated for 1 h in 10% normal goat serum (in PBS) to block for non-specific secondary antibody binding (ThermoFisher, 16210–072). Sections were incubated with primary antibodies (Table 2) diluted in 1% normal goat serum overnight at 4°C in a humidified slide chamber. Sections were subsequently washed in PBS (3 × 5 min). Secondary antibodies (Table 3), diluted in 1% normal goat serum with Hoechst 33342 nuclear counterstain (1:20,000; ThermoFisher, H1399), were incubated on sections for 3 h at room temperature. Slides were washed in PBS (3 × 5 min) and coverslipped (Menzel-Gläser; #1.5) using Prolong^®^ Diamond Antifade Mountant (ThermoFisher). Coverslips were sealed with nail polish and slides were stored at 4 °C, protected from light.

**Table 3:** Secondary antibodies used for immunohistochemistry.

Antibody	Target	Species	Isotype	Manufacturer	Catalogue No.	Dilution
Goat anti-mouse AlexaFluor^®^ 488	Mouse	Goat	IgG (H+L)	ThermoFisher	A11001	1:500
Goat anti-mouse AlexaFluor^®^ 488	Mouse	Goat	IgG1	ThermoFisher	A21121	1:500
Goat anti-mouse AlexaFluor^®^ 488	Mouse	Goat	IgG2a	ThermoFisher	A21131	1:500
Goat anti-rabbit AlexaFluor^®^ 488	Rabbit	Goat	IgG (H+L)	ThermoFisher	A11034	1:500
Goat anti-chicken AlexaFluor^®^ 488	Chicken	Goat	IgG (H+L)	ThermoFisher	A11039	1:500
Goat anti-mouse AlexaFluor^®^ 594	Mouse	Goat	IgG (H+L)	ThermoFisher	A11032	1:500
Goat anti-mouse AlexaFluor^®^ 594	Mouse	Goat	IgG1	ThermoFisher	A21125	1:500
Goat anti-mouse AlexaFluor^®^ 594	Mouse	Goat	IgG2a	ThermoFisher	A21135	1:500
Goat anti-rabbit AlexaFluor^®^ 594	Rabbit	Goat	IgG (H+L)	ThermoFisher	A11037	1:500
Goat anti-chicken AlexaFluor^®^ 594	Chicken	Goat	IgG (H+L)	ThermoFisher	A21449	1:500
Goat anti-mouse AlexaFluor^®^ 647	Mouse	Goat	IgG2c	Jackson ImmunoResearch	115-607-188	1:500
Goat anti-guinea pig AlexaFluor^®^ 647	Guinea Pig	Goat	IgG (H+L)	ThermoFisher	A21450	1:500
Goat anti-rabbit AlexaFluor^®^ 647	Rabbit	Goat	IgG (H+L)	ThermoFisher	A21245	1:500
Goat anti-chicken AlexaFluor^®^ 647	Chicken	Goat	IgG (H+L)	ThermoFisher	A21449	1:500
Goat anti-rabbit AlexaFluor^®^ 800	Rabbit	Goat	IgG (H+L)	LI-COR	926-32211	1:500
Donkey anti-sheep AlexaFluor^®^ 647	Sheep	Donkey	IgG (H+L)	ThermoFisher	A21448	1:500

All antibodies were optimised using a variety of antigen retrieval methods. For heat-induced antigen retrieval, both citrate buffer (pH 6.0) and TRIS-EDTA (pH 9.0) buffer were investigated (both with and without detergent). We did not observe any major differences between the two buffer types, however, TRIS-EDTA (pH 9.0) afforded a slightly higher level of detection across all of the α-Syn antibodies investigated; antigenicity was slightly improved with the use of detergent (0.05% Tween 20). The use of formic acid substantially increased the antigenicity of all α-Syn antibodies investigated. All immunohistochemical interrogations in this study included validations with no-primary controls, in which primary antibodies were not added. These validations were conducted to control for background autofluorescence signatures and any non-specific antibody labelling. Any autofluorescence signature fell below the segmentation threshold of interrogated aggregate morphologies.

To ensure that the observed epitope-specific immunolabelling was not the result of steric hindrance caused by competing differential antibody-epitope affinities, we conducted a series of progressive single-label antibody validations. Briefly, one section from each brain region was simultaneously immunolabelled with the full panel of α-Syn antibodies. The adjacent section, meanwhile, was only immunolabelled with the MJFR1 α-Syn antibody. Sections were imaged using confocal microscopy and Z-stack images of several LNs and LBs were acquired. Each single-labelled section was then immunolabelled with the NAC-specific antibody and the same aggregates were imaged as they had been previously. This sequential immunolabel-image procedure was then repeated for the pS129 antibody and then the N-terminus antibody. No detectable differences in epitope-specific immunoreactivities were observed between the simultaneously immunolabelled sections and the serially immunolabelled sections, thereby mitigating steric hindrance as a confounding influence.

### Proteolytic digestion assay

To characterise the aggregation state of the various α-Syn aggregate morphologies observed, we conducted time-dependent proteolysis by means of proteinase K digestion. Hippocampus (*n* = 8) and substantia nigra (*n* = 8) sections were immunolabelled as described above and confocal images of representative aggregates were acquired. Sections were subsequently de-coverslipped and washed to remove residual mountant media. Sections were then treated with proteinase K^15^ (7.6 μg/mL) at 37 °C for 30 min, after which sections were washed and re-labelled for a second round of image acquisition. This process was repeated with proteinase K being added for an additional 10 min each round to give 40, 50, and 60 min (cumulative) time points. In each round of imaging, the same representative aggregates were acquired for all confocal image acquisitions. For whole-tissue image acquisitions, the same region of interest was acquired in each successive acquisition round. All epitope-specific quantifications were internally normalised to pre-treatment (no proteinase K) values to allow each successive timepoint to be presented as a relative percentage of the pre-treatment value.

### Image acquisition and quantification

Confocal images were acquired using an LSM 800 with Airyscan confocal microscope (Zeiss, Germany) with a 63x/1.4 NA Plan Apochromat DIC M27 oil immersion objective lens and GaAsp-PMT detector. Images were acquired using the built-in Airyscan module and processed using the ZEN microscopy software (Zeiss). All images were acquired using optimal Nyquist sampling parameters and those acquired in a Z-series used the optimal step size of 0.13 μm. Images were specifically acquired from the anterior olfactory nucleus in the olfactory bulb, the dorsal motor nucleus of the vagus nerve in the medulla, the substantia nigra pars compacta in the midbrain, the middle temporal and middle frontal gyrus of the cortex, the CA2 region of the hippocampus proper and the entorhinal cortex. TMA cores and all whole-section images were imaged using an automated fluorescence microscope (Zeiss Z2 Axioimager) equipped with a MetaSystems VSlide slide scanner (MetaSystems) running MetaFer (V 3.12.1) with a 20x/0.9 NA dry objective lens. Images were stitched using MetaCyte software. Following image acquisition, the total section scans were viewed using VSViewer (V 2.1.132; MetaSystems). Regions of interest were identified and extracted for subsequent analysis in FIJI/ImageJ.

All downstream quantitative image analysis was performed using FIJI/ImageJ (V 2.3.0/1.53q). Briefly, background signal intensity was subtracted using an automated Gaussian blur algorithm and pathological α-Syn labelling was automatically thresholded using the Otsu algorithm for each epitope-specific antibody. Accurate aggregate segmentation was manually confirmed prior to conducting downstream analysis. The total α-Syn immunolabelling area was determined by calculating the net α-Syn immunolabelling across the N-terminus (849102), pS129 (Ab184674), and C-terminus (Ab138501) antibodies. Unique epitope-specific α-Syn immunolabelling area was determined for each antibody by subtracting the net thresholded area of the other two antibodies from the total α-Syn immunolabelling area. The net overlapping immunolabelling area was determined by subtracting all epitope-specific antibody immunolabelling areas from the total α-Syn immunolabelling area. Image deconvolution was performed using the Zeiss Zen Blue software package, with the built in Airyscan processing module; all images were processed using the 2D/3D standard deconvolution settings, depending on whether images were single or multiple planes.

### Western blot analysis

Samples for western blot analysis were denatured in NuPage LDS sample buffer (ThermoFisher Scientific; NP0007) at 95 °C for 10 min. Samples were loaded into 4–12% Bis-Tris gels (ThermoFisher Scientific; NP0336BOX) to be resolved via SDS-PAGE, in accordance with the manufacturers protocol. Gels were run using an MES SDS running buffer (ThermoFisher Scientific; NP0002) with a Spectra^™^ Multicolor Low Range Protein Ladder (ThermoFisher Scientific; 26628), to facilitate the resolve of small molecular weight proteins. Gels were run for 30 min, applying a constant voltage of 175 V. Once protein migration was complete, gels were transferred using a MiniBlot Module (ThermoFisher Scientific; B1000) with NuPage transfer buffer (ThermoFisher Scientific; NP0006), in accordance with the manufacturers protocol. Gels were transferred onto PVDF membranes (Millipore, IPFL00005), which had been pre-activated in 100% methanol for 2 min, for 1 h using a constant voltage of 20 V. Membranes were blocked for 1 h at room temperature in a 1:2 solution of Intercept^®^ (TBS) Blocking Buffer (LI-COR, 927–60001) and TBS-T (TBS with 0.01% Tween 20). Primary antibodies (Table 2) were incubated overnight at 4 °C in blocking buffer. Membranes were washed in TBS-T (3 × 10 min) and incubated with secondary antibodies (diluted 1:10,000 in blocking buffer with 0.02% SDS) for 3 h at room temperature, protected from light. Membranes were washed in TBS-T (3 × 10 min), before being washed a final time for 10 min in TBS. Image acquisition was performed using a BioRad ChemiDoc^TM^ MP Imaging System. All blots derive from the same experiment and were processed in parallel.

### Statistical analysis

Data visualisation and statistical analysis were performed using RStudio (R version 4.2.1). All data are presented as mean ± standard deviation (SD). The Shapiro-Wilk test was used to assess data distribution. Linear regression, using Pearson’s correlation coefficient, was used to analyse correlations between epitope-specific α-Syn antibody labelling. One-way analysis of variance (ANOVA) was used to compare differential epitope-specific α-Syn immunoreactivities both within and across interrogated brain regions, with Tukey’s multiple comparison adjustment. Statistical significance was set as *p* < 0.05.

### Reporting summary

Further information on research design is available in the Nature Research Reporting Summary linked to this article.

### Supplementary information

Supplementary Material

Reporting Summary

## Data Availability

All data are available in the main text or Supplementary Materials. The raw data that support the findings of this study are available from the corresponding author upon request.
