# Supplementary material for: Aggregate-prone brain regions in Parkinson’s disease are rich in unique N-terminus α-synuclein conformers with high proteolysis susceptibility
Source: NPJ Parkinsons Dis. 2024 Jan 2;10:1. doi: 10.1038/s41531-023-00614-w (PMC10762179; doi:10.1038/s41531-023-00614-w)
Supplement: Supplementary file 1 — Supplementary Material [file 41531_2023_614_MOESM1_ESM.pdf]

Supplementary Material

Supplementary Tables

Supplementary Table 1. Mean unique epitope-specific N-terminus immunolabelling and α-Syn pathology load across PD cases and regions.

Region	Mean Epitope-specific N-terminus Immunolabelling (%)	Pathology Load (%)
Olfactory Bulb	38 ± 9	1.82 ± 1.42
Medulla	21 ± 10	3.23 ± 1.66
Substantia Nigra	27 ± 12	3.19 ± 2.18
Hippocampus (CA2)	23 ± 7	4.55 ± 4.39
Entorhinal Cortex	40 ± 14	2.50 ± 2.31
Middle Temporal Gyrus	34 ± 20	0.85 ± 0.27
Middle Frontal Gyrus	34 ± 20	0.71 ± 0.52

Supplementary Table 2. Case information for cases used in tissue microarray analysis. PMD, post-mortem delay; PD, Parkinson's disease; CLBD, cortical Lewy body disease; AD, Alzheimer's disease.

Case	Neuropathological Diagnosis	Age	Sex	PMD (hrs)	Cause of Death	Duration with Disease (years)
PD30	PD/CLBD (neocortical/diffuse)	82	M	19	Multiple organ failure	11
PD31	PD	67	M	25	Respiratory failure	5
PD32	PD/CLBD	71	M	8	Ischaemic heart disease/congestive heart failure	25
PD35	CLBD (diffuse)	73	M	16	Pneumonia	15
PD36	PD	78	F	22.5	Bronchopneumonia/cardiovascular disease	–
PD37	PD/CLBD (neocortical/diffuse)	81	M	4	End stage Parkinson's disease	13
PD41	PD/AD	81	M	13	Renal failure/urinary sepsis	1
PD43	CLBD	60	F	15.5	Bronchopneumonia/multisystem organ failure	7
PD50	PD/CLBD	88	M	6	Myocardial infarction/ischaemic heart disease	20
PD52	PD/CLBD (neocortical/diffuse)	84	M	5	Acute myocardial infarction	12
PD56	PD/CLBD (neocortical/diffuse)	74	M	10.5	End stage Lewy body disease	12
PD57	CLBD/AD (neocortical/diffuse)	90	F	14	Bronchopneumonia	–
PD60	PD/CLBD/AD (neocortical/diffuse)	80	M	18	Urosepsis	26
PD63	PD/CLBD/AD (neocortical/diffuse)	91	F	5	Parkinson's disease	22
PD65	PD/CLBD (neocortical/diffuse)	67	M	2.25	End stage Parkinson's disease and dementia with Lewy bodies	9
PD66	PD/CLBD (neocortical/diffuse)	73	M	17.5	Aspiration pneumonia	22
PD67	PD/CLBD (neocortical)	65	M	17	Bronchopneumonia	12
PD69	PD/CLBD (limbic)	84	F	22	End stage Parkinson's disease	–
PD71	PD/CLBD (limbic)	80	M	5.5	Pneumonia	9
PD73	PD/CLBD (neocortical)	83	F	4	Pneumonia/end stage Parkinson's disease	–
PD77	PD/CLBD (limbic)	76	F	6.5	Intra-abdominal high grade serous carcinoma	23
PD78	PD/CLBD (neocortical)	80	M	5.5	End stage Parkinson's disease	–
PD79	PD/CLBD (neocortical)	77	M	6.5	Severe LB dementia, cognitive loss, Loss oral intake	22

H180	Normal	73	M	33	Ischaemic heart disease	N/a
H184	Normal	35	M	20	Electrocution	N/a
H186	Normal	68	M	21	Ischaemic heart disease	N/a
H187	Normal	98	F	15	Caecal carcinoma	N/a
H189	Normal	41	M	16	Asphyxia	N/a
H190	Normal	72	F	19	Ruptured myocardial infarction	N/a
H191	Normal	77	M	25	Ischaemic heart disease	N/a
H194	Normal	68	M	22.5	Coronary atherosclerosis	N/a
H204	Normal	66	M	9	Ischaemic heart disease	N/a
H209	Normal	48	M	23	Ischaemic heart disease	N/a
H226	Normal	73	F	48	Mesothelioma	N/a
H227	Normal	78	F	4	Cerebrovascular accident	N/a
H228	Normal	87	F	21	Ischaemic heart disease	N/a
H231	Normal	65	M	8	Ischaemic heart disease	N/a
H237	Normal	81	M	17	Acute bacterial endocarditis	N/a
H238	Normal	63	F	16	Dissecting aortic aneurysm	N/a
H239	Normal	64	M	15.5	Ischaemic heart disease	N/a
H242	Normal	61	M	19.5	Coronary atherosclerosis	N/a
H243	Normal	77	F	13	Ischaemic heart disease/coronary atherosclerosis	N/a
H244	Normal	76	M	16	Ischaemic heart disease/coronary atherosclerosis	N/a
H245	Normal	63	M	20	Asphyxia	N/a
H246	Normal	89	M	17	Type II myocardial infarction	N/a
H247	Normal	51	M	31	Bilateral pulmonary thromboembolism secondary to right calf deep vein thrombosis	N/a
H250	Normal	93	F	19	Acute coronary syndrome; pneumonia	N/a

Supplementary Figures

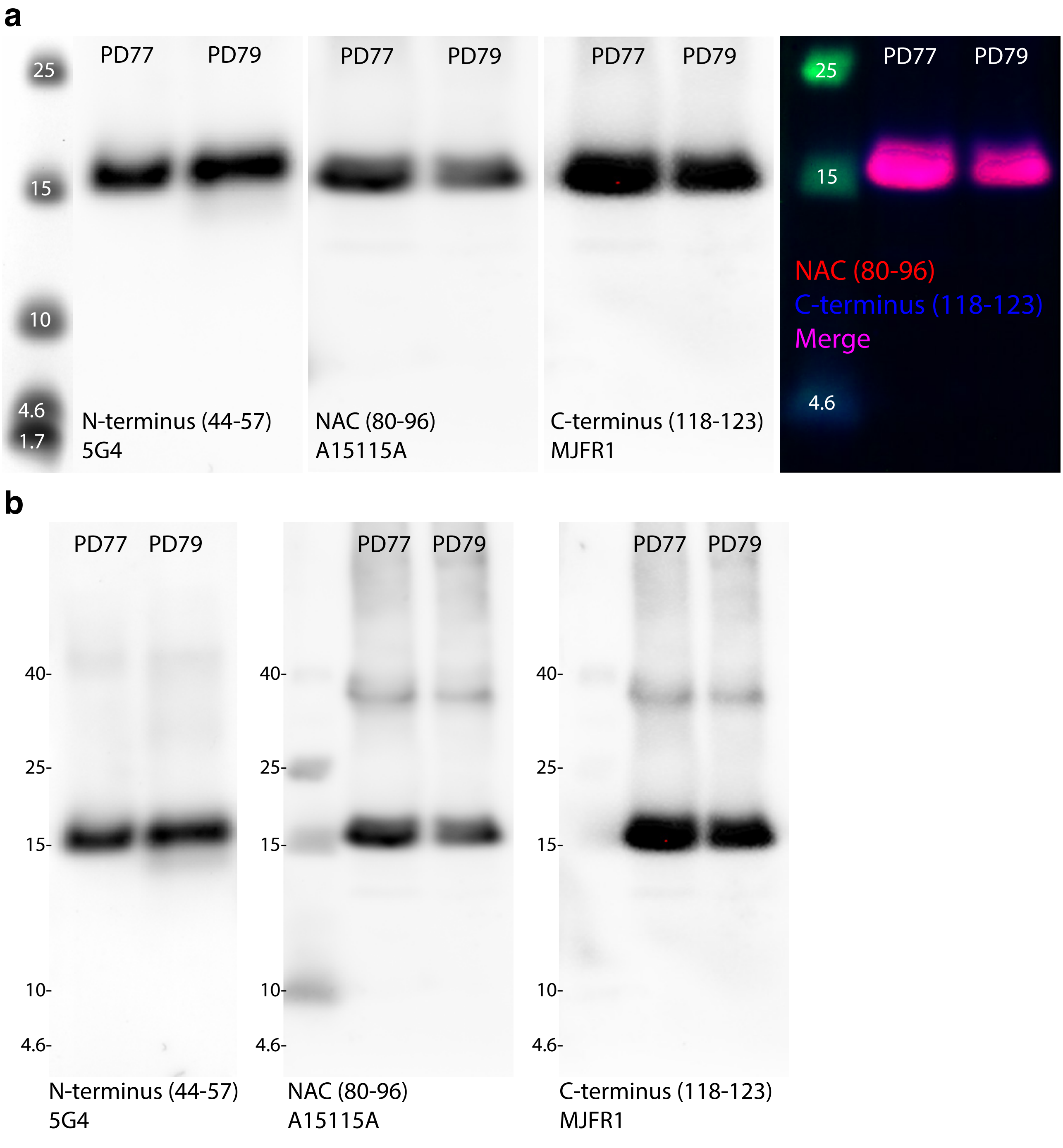

Supplementary Figure 1. Western blots validating the α-Syn specificity of the N-terminus (44-57), NAC (80-96), and MJFR1 (118-123) antibodies. a Each antibody exhibited a clear band at ~15kDa when immunolabelling substantia nigra tissue homogenates from two PD brains (PD77 and PD79). The additional fluorescence acquisition (right) demonstrates the duplex immunolabelling of both the NAC (80-96; red) and MJFR1 (118-123; blue) antibodies, adjacent to the protein standard. This has been included for quality assurance. All blots derive from the same experiment and were processed in parallel. **b** Full uncropped western blots for all acquisitions shown in **a**.

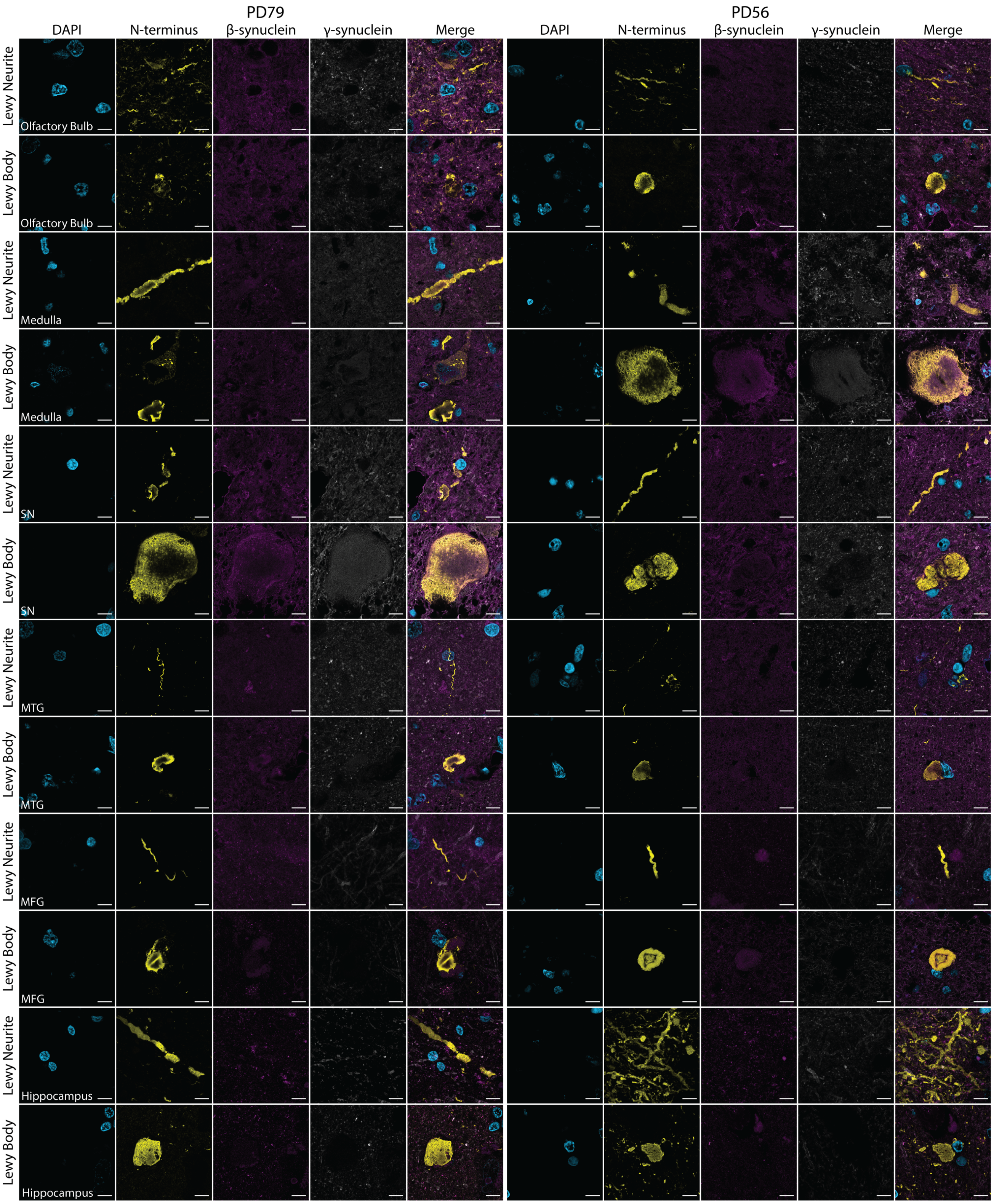

Supplementary Figure 2. Representative confocal immunofluorescence images from PD79 *(left)* and PD56 *(right)* validating that the N-terminus α-Syn antibody does not non-specifically immunolabel β-synuclein or γ-synuclein. SN, substantia nigra; MTG, middle frontal gyrus; MFG, middle frontal gyrus. Scale bar, 10μm.

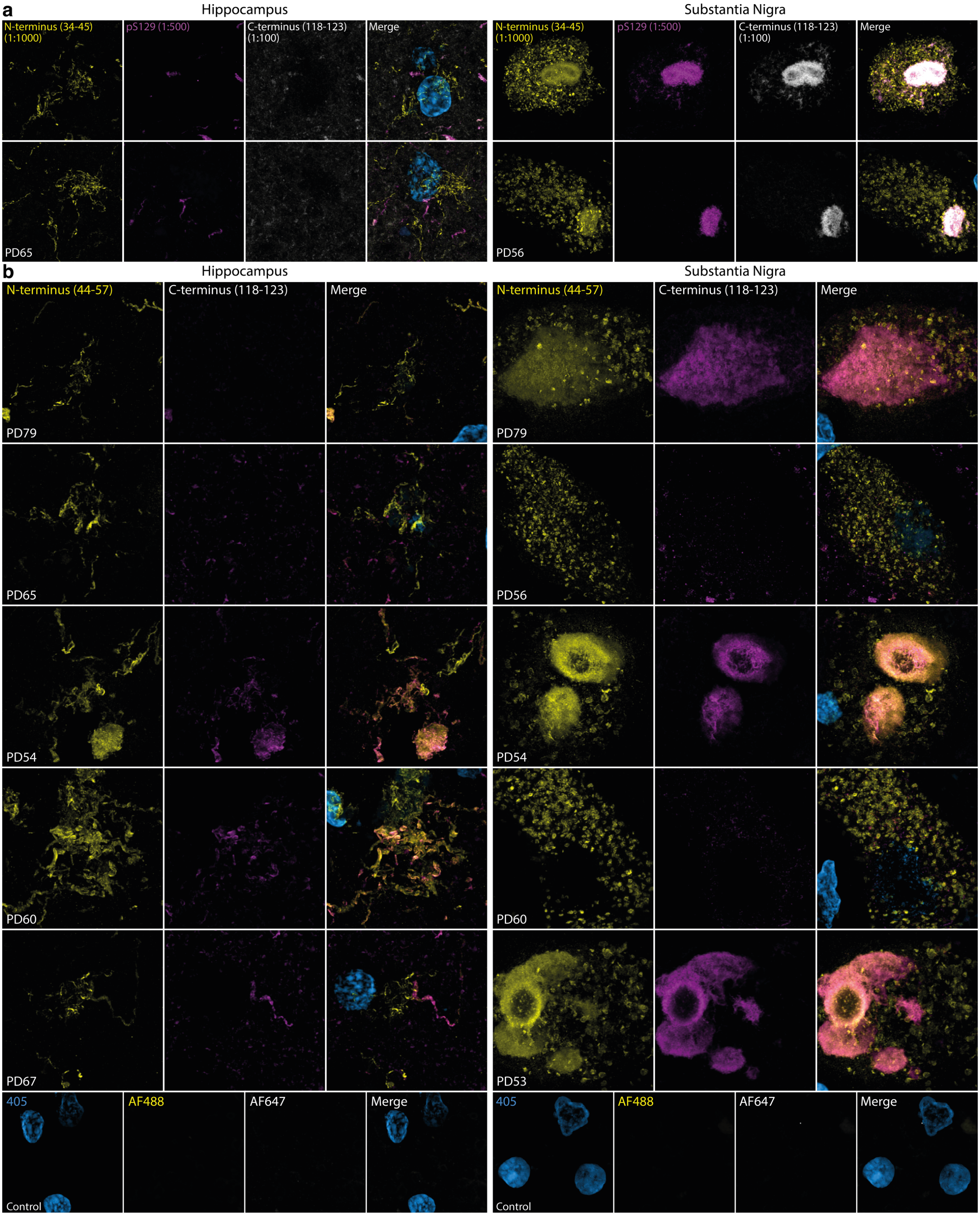

Supplementary Figure 3. Representative confocal immunofluorescence images validating the affinity and specificity of the N-terminus (34-45) antibody. a Representative images validating the absence of pS129 and C-terminus (118-123) epitope detection in exclusively N-terminus immunoreactive glial and neuronal lysosomal α-Syn aggregates. Both the pS129 and C-terminus antibodies were immunolabelled at saturated concentrations (1:500, and 1:100, respectively); this was 8 times the optimal pS129 (Ab184674) concentration, and 10 times the optimal C-terminus (Ab138501) concentration. **b** Representative images depicting epitope-specific N-terminus (44-57) α-Syn immunolabelling in both glial and neuronal lysosomal α-Syn aggregates in 7 PD cases. Bottom row displays image acquisitions of PD tissue sections in which the primary antibodies were not added.

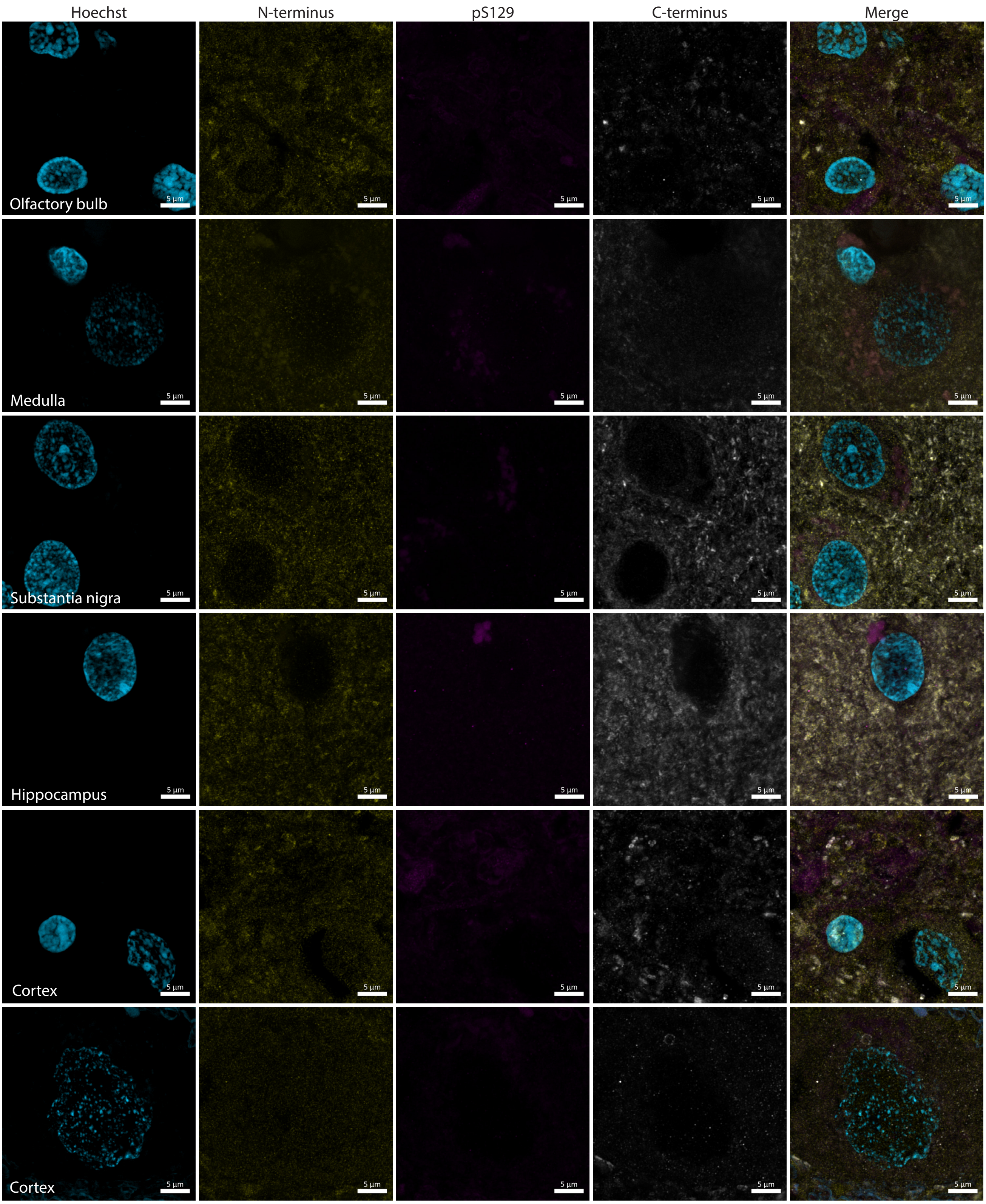

Supplementary Figure 4. Representative confocal immunofluorescence images demonstrating the immunolabelling profile of α-Syn antibodies in neurologically normal (control) cases. Scale bar, 5µm.

a

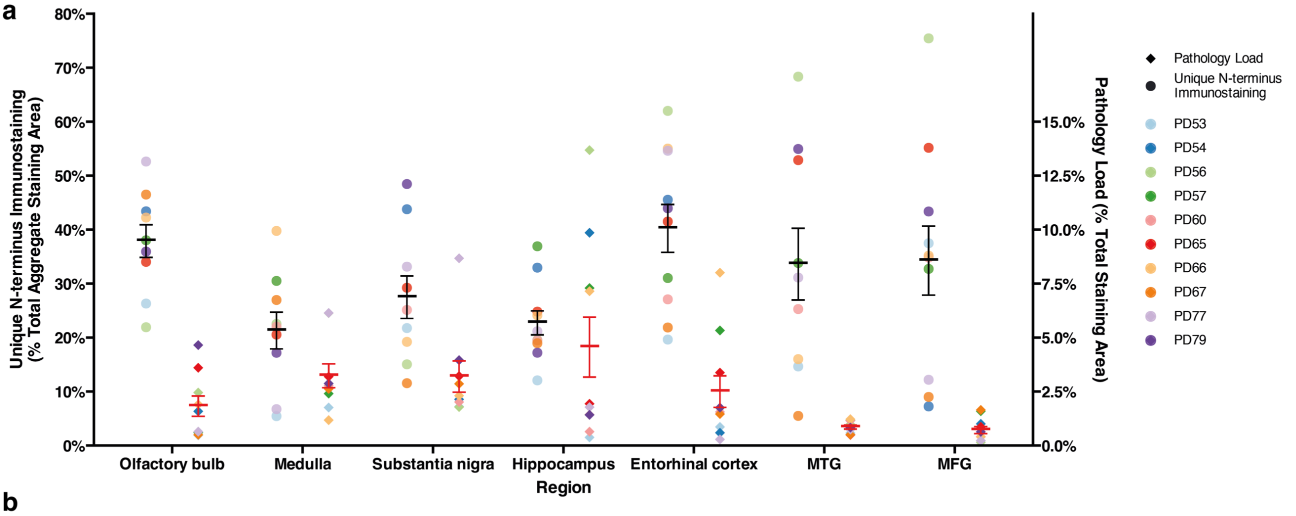

b

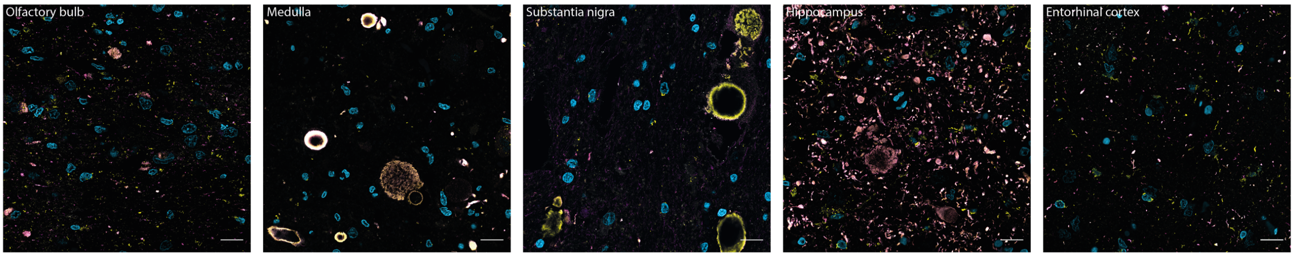

Supplementary Figure 5. Quantification of epitope-specific N-terminus α-Syn immunolabelling in pathologically significant whole-tissue sections of PD cases. a Scatter plot demonstrating the epitope-specific N-terminus immunolabelling (circles) in whole-tissue sections for each PD case as a percentage of the total pathological α-Syn immunolabelling area. The α-Syn pathology load (diamonds) for each case is also shown on the secondary axis. Black crossbars and error bars present the mean epitope-specific N-terminus α-Syn immunolabelling (± SD) for each region. Red crossbars and error bars present the mean pathology load (± SD) for each region. **b** Representative images from the olfactory bulb, medulla, substantia nigra, hippocampus, and entorhinal cortex (yellow, N-terminus; magenta, pS129; cyan, C-terminus). Scale bar, 20μm.

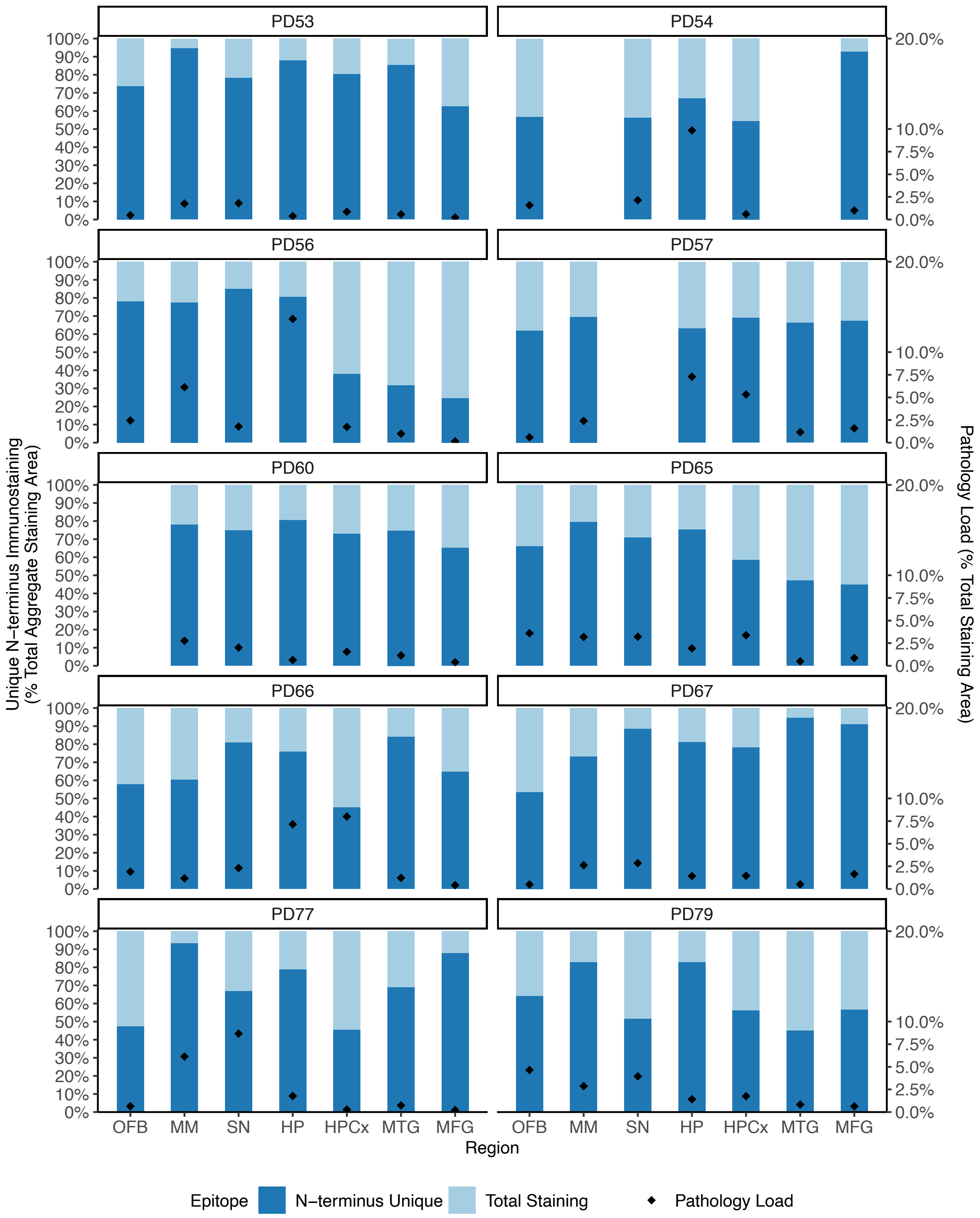

Supplementary Figure 6. Proportion of total α-Syn aggregate immunolabelling that exhibits exclusive N-terminus immunoreactivity in pathologically significant regions of the human brain with PD. α-Syn pathology load (black diamonds, secondary axis) is normalised to tissue area. OFB, olfactory bulb; MM, middle medulla; SN, substantia nigra; HP hippocampus (CA2); HPCx, entorhinal cortex; MTG, middle temporal gyrus; MFG, middle frontal gyrus.

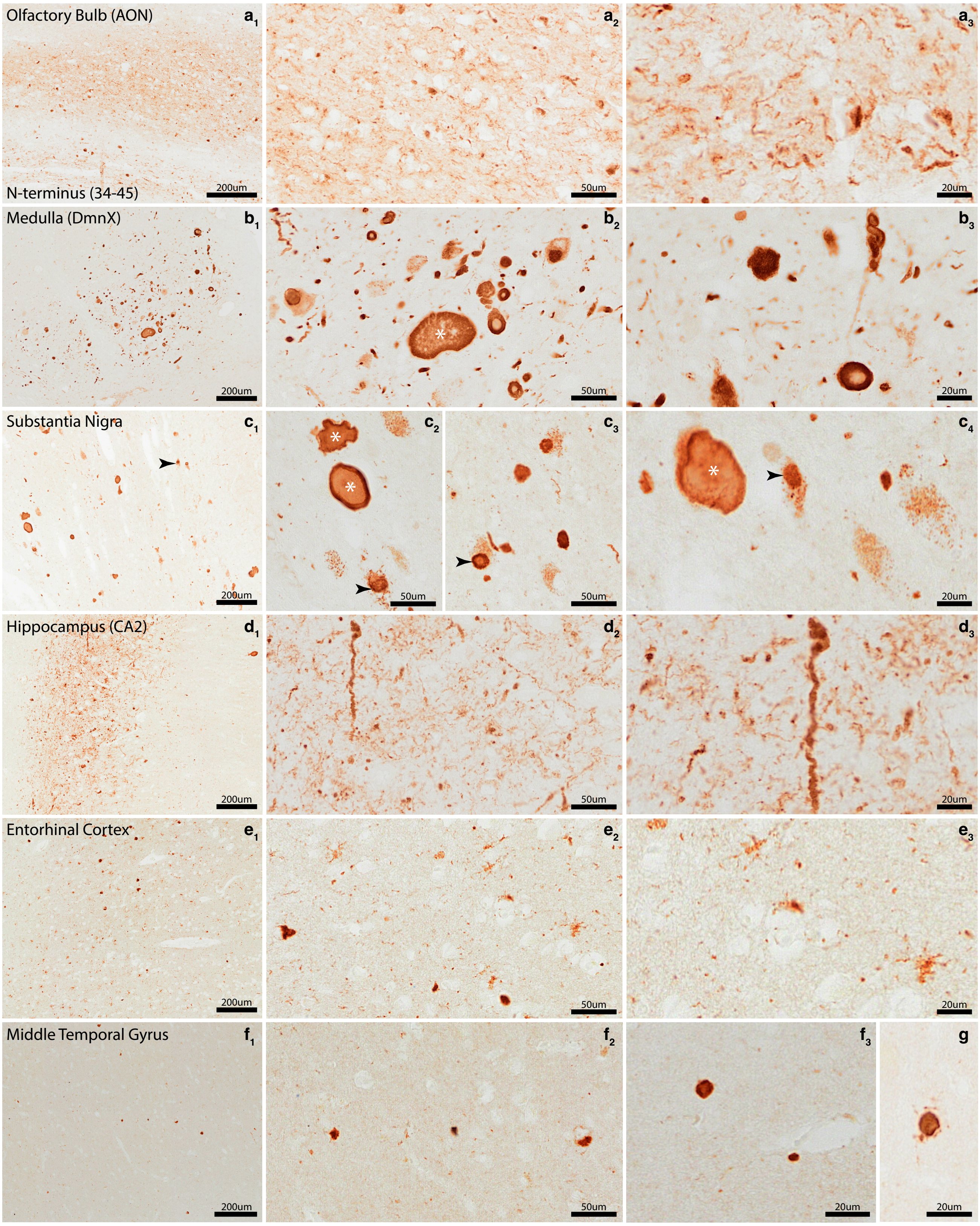

Supplementary Figure 7. Anatomical and morphological distribution of N-terminus (34-45) immunopositive α-Syn aggregates in PD. a Small-medium thread-like Lewy neurites with small interspersed Lewy bodies are prominent within the AON of the olfactory bulb. **b** A mixture of small thread-like Lewy neurites and large club-shaped Lewy neurites predominate in the DmnX. Several large extracellular Lewy bodies (*B_2,* white asterisk) are also common. **c** Both thread-like and club-like Lewy neurites are common within the substantia nigra pars compacta. In addition, much larger Lewy bodies, both intracellular (black arrowheads) and extracellular (white asterisks), are prominent. Extracellular Lewy bodies do not localise with any surrounding neuromelanin, as is the case for intracellular Lewy bodies. **d** A dense web of Lewy neurites spanning the CA2 subregion of the hippocampus proper is characteristic within the hippocampus. **e** Small Lewy bodies and glial-

like aggregates are diffuse within the entorhinal cortex. **f**, **g** Lewy pathology within the wider cortical regions is relatively sparse and evenly distributed between small Lewy bodies and thread-like Lewy neurites. AON, anterior olfactory nucleus; DmnX, dorsal motor nucleus of the vagus nerve.

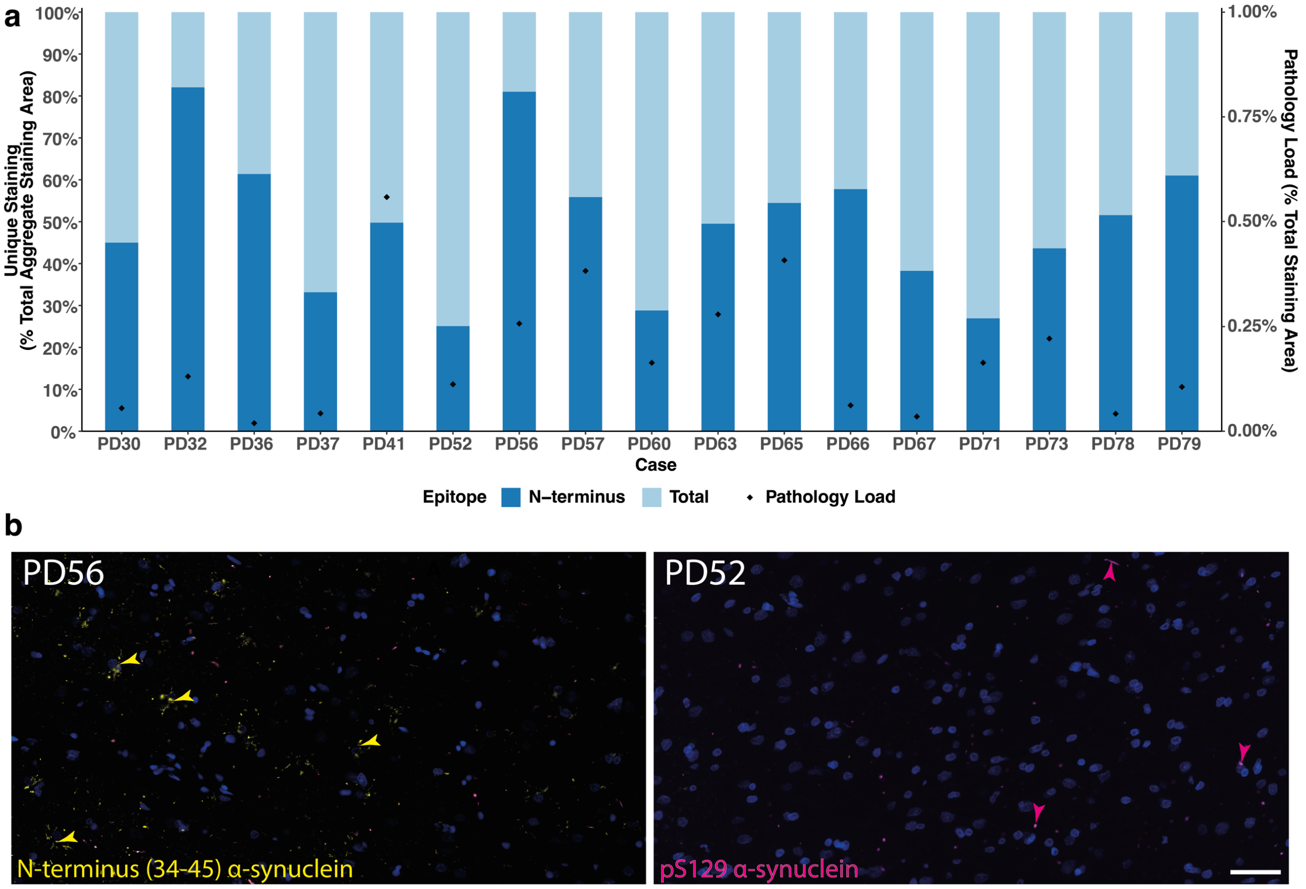

Supplementary Figure 8. Quantification of epitope-specific N-terminus α-Syn immunolabelling in a tissue microarray of the PD MTG. a Bar plot quantifying the epitope-specific N-terminus immunolabelling for each PD case (n = 17) in a tissue microarray of the human MTG. Data is normalised to the total α-Syn immunolabelling area for each individual case. The α-Syn pathology load (black diamonds) of each case is shown on the secondary axis. Data is presented as mean ± SD. **b** Representative images from a case with high epitope-specific N-terminus immunolabelling (yellow arrowheads) and low epitope-specific pS129 immunolabelling (PD56), and a case with high epitope-specific pS129 immunolabelling (magenta arrowheads) and low epitope-specific N-terminus labelling (PD52). Scale bar, 50μm.

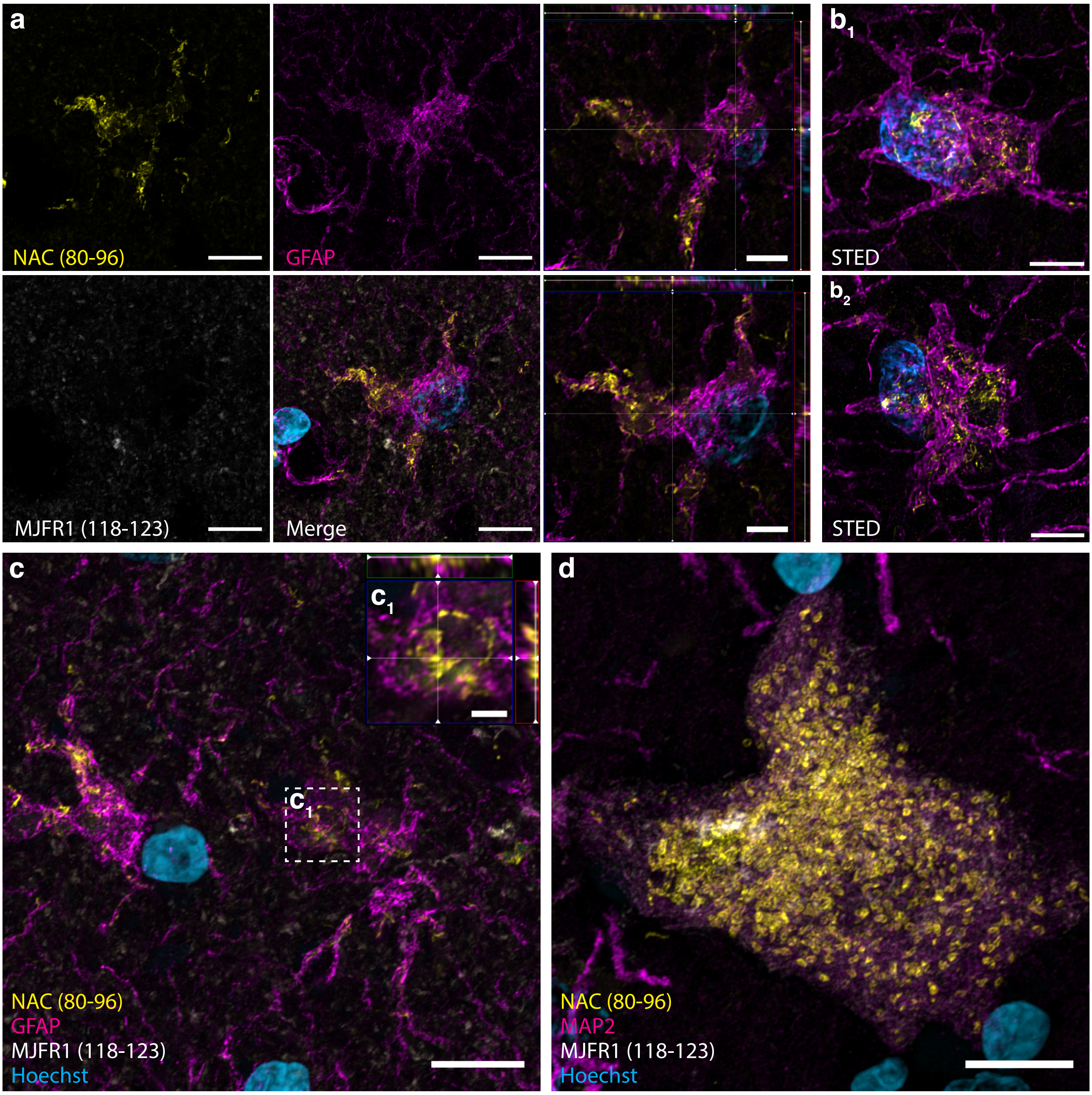

Supplementary Figure 9. Representative confocal and STED immunofluorescence images validating the glial and lysosomal localisation of epitope-specific NAC (80-96) α-Syn immunolabelling. a-c Astrocytic (magenta, GFAP) localisation of NAC (80-96) α-Syn (yellow). Dashed white box indicates the region sampled for higher magnification image acquisition in **c_1** (scale bar, 1μm). **d** Representative confocal image validating the neuronal localisation (magenta, MAP2) of NAC (80-96) α-Syn (yellow). Scale bar, 5μm.

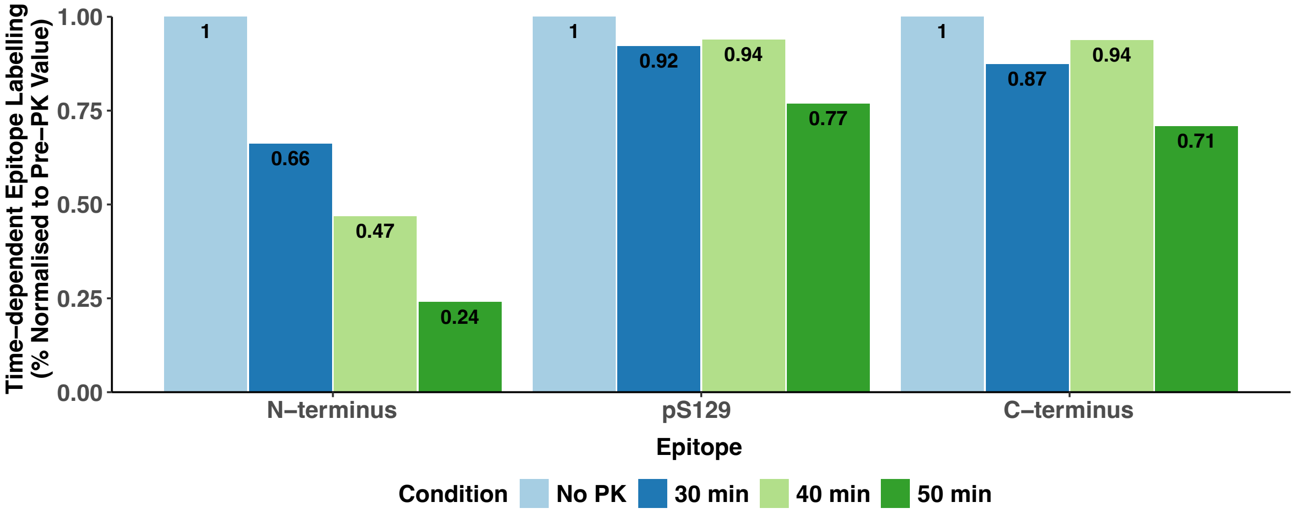

Supplementary Figure 10. **Quantification of epitope-specific α-Syn immunolabelling in whole-section image acquisitions following time-dependent proteolytic digestion with proteinase K.** All quantifications are normalised to the pre-treatment immunolabelling values. The N-terminus epitope progressively degraded over time, whereas the pS129 and C-terminus (118-123) epitopes remained relatively intact.
