# Supplementary material for: Aggregate-prone brain regions in Parkinson’s disease are rich in unique N-terminus α-synuclein conformers with high proteolysis susceptibility
Source: NPJ Parkinsons Dis. 2024 Jan 2;10:1. doi: 10.1038/s41531-023-00614-w (PMC10762179; doi:10.1038/s41531-023-00614-w)
Supplement: Supplementary file 2 — Reporting Summary [file 41531_2023_614_MOESM2_ESM.pdf]

nature portfolio

Corresponding author(s): Maurice A Curtis

Last updated by author(s): Nov 28, 2023

Reporting Summary

Nature Portfolio wishes to improve the reproducibility of the work that we publish. This form provides structure for consistency and transparency in reporting. For further information on Nature Portfolio policies, see our Editorial Policies and the Editorial Policy Checklist.

Statistics

For all statistical analyses, confirm that the following items are present in the figure legend, table legend, main text, or Methods section.

n/a	Confirmed	
☐	☒	The exact sample size (*n*) for each experimental group/condition, given as a discrete number and unit of measurement
☒	☐	A statement on whether measurements were taken from distinct samples or whether the same sample was measured repeatedly
☐	☒	The statistical test(s) used AND whether they are one- or two-sided<br>*Only common tests should be described solely by name; describe more complex techniques in the Methods section.*
☒	☐	A description of all covariates tested
☐	☒	A description of any assumptions or corrections, such as tests of normality and adjustment for multiple comparisons
☐	☒	A full description of the statistical parameters including central tendency (e.g. means) or other basic estimates (e.g. regression coefficient) AND variation (e.g. standard deviation) or associated estimates of uncertainty (e.g. confidence intervals)
☒	☐	For null hypothesis testing, the test statistic (e.g. F, t, r) with confidence intervals, effect sizes, degrees of freedom and P value noted<br>*Give P values as exact values whenever suitable.*
☒	☐	For Bayesian analysis, information on the choice of priors and Markov chain Monte Carlo settings
☒	☐	For hierarchical and complex designs, identification of the appropriate level for tests and full reporting of outcomes
☒	☐	Estimates of effect sizes (e.g. Cohen's *d*, Pearson's *r*), indicating how they were calculated

Our web collection on statistics for biologists contains articles on many of the points above.

Software and code

Policy information about availability of computer code

Data collection	Images were stitched using MetaCyte software. Following image acquisition, the total section scans were viewed using VSViewer (V 2.1.132; MetaSystems) software.
Data analysis	All quantitative image analysis was performed using FIJI/ImageJ (V 2.3.0/1.53q). Data visualisation and statistical analysis were performed using RStudio (R version 4.2.1).

For manuscripts utilizing custom algorithms or software that are central to the research but not yet described in published literature, software must be made available to editors and reviewers. We strongly encourage code deposition in a community repository (e.g. GitHub). See the Nature Portfolio guidelines for submitting code & software for further information.

Data

Policy information about availability of data

All manuscripts must include a data availability statement. This statement should provide the following information, where applicable:

- Accession codes, unique identifiers, or web links for publicly available datasets
- A description of any restrictions on data availability
- For clinical datasets or third party data, please ensure that the statement adheres to our policy

All data are available in the main text or Supplementary Materials. The raw data that support the findings of this study are available from the corresponding author upon reasonable request.

Research involving human participants, their data, or biological material

Policy information about studies with human participants or human data. See also policy information about sex, gender (identity/presentation), and sexual orientation and race, ethnicity and racism.

Reporting on sex and gender	There is no specific reporting on sex and/or gender. Details of the sex of post-mortem cases used in this study are displayed in Table 1 and Supplementary Table S2.
Reporting on race, ethnicity, or other socially relevant groupings	There is no specific reporting on race, ethnicity, or other socially relevant groupings.
Population characteristics	All patient samples utilised in the present study were post-mortem human brain samples. Full details of post-mortem cases used in this study are displayed in Table 1 and Supplementary Table S2. Any additional information has been excluded for confidentiality reasons.
Recruitment	The neurologically normal cases (n = 24) had no clinical history of neurological abnormalities, and no other significant neuropathology was noted upon post-mortem examination. The mean age (± standard deviation) of normal cases was 71 ± 16 and ranged from 35–98 years. The mean post-mortem delay of normal cases was 20 ± 9 hours with a range of 4–48 hours. All PD cases (n = 25) had a clinical history of Parkinson's disease, and pathological features were consistent with PD pathology as confirmed by a neuropathologist. Parkinson's disease cases had a disease duration ranging from 1–25 years, with the mean duration being 14 years. The mean age of Parkinson's disease cases was 78 ± 7 and ranged from 60–91 years; the mean post-mortem delay was 12 ± 7 hours with a range of 2.25–25 hours.
Ethics oversight	The studies were approved by the NZ Health 124 and Disabilities Ethics Committee and by the University of Auckland Human Participants Ethics 125 Committee (Ref: 011654).

Note that full information on the approval of the study protocol must also be provided in the manuscript.

Field-specific reporting

Please select the one below that is the best fit for your research. If you are not sure, read the appropriate sections before making your selection.

☒ Life sciences ☐ Behavioural & social sciences ☐ Ecological, evolutionary & environmental sciences

For a reference copy of the document with all sections, see nature.com/documents/nr-reporting-summary-flat.pdf

Life sciences study design

All studies must disclose on these points even when the disclosure is negative.

Sample size	Sample size was determined based on disease/region availability. We utilised the olfactory bulb, medulla, substantia nigra, hippocampus, entorhinal cortex, middle temporal gyrus and middle frontal gyrus of (n = 10) human brains with Parkinson's disease. To ensure these results were replicated in a larger sample size, we utlised a tissue microarray containing middle temrpal gyrus cores from (n = 23) Parkinson's disease brains and (n = 24) neurologically normal brains. Full details of sample size and sample characteristics can be viewed in Table 1 and Supplementary Table S2.
Data exclusions	No data was excluded from analysis.
Replication	We conducted 3 separate repeats of the tissue microarray analysis detailed in the results section "Differential epitope-specific α-synuclein immunolabelling within the middle temporal gyrus" and all repeats yielded the same result.
Randomization	Randomization was not relevant for our study as we were utilising post-mortem tissue samples that had been neuropathologically confirmed by a neuropathologist.
Blinding	Blinding was not relevant to the present study as comparisons of epitope-specific antibody immunoreactivities were internally normalised within each individual case/region.

Reporting for specific materials, systems and methods

We require information from authors about some types of materials, experimental systems and methods used in many studies. Here, indicate whether each material, system or method listed is relevant to your study. If you are not sure if a list item applies to your research, read the appropriate section before selecting a response.

Materials & experimental systems

n/a	Involved in the study	
☐	☒	Antibodies
☒	☐	Eukaryotic cell lines
☒	☐	Palaeontology and archaeology
☒	☐	Animals and other organisms
☒	☐	Clinical data
☒	☐	Dual use research of concern
☒	☐	Plants

Methods

n/a	Involved in the study	
☒	☐	ChIP-seq
☒	☐	Flow cytometry
☒	☐	MRI-based neuroimaging

Antibodies

Antibodies used	Details of all antibodies used in this study are included in Table 2 and Table 3, as requested.
Validation	All relevant information has been included in our manuscript.
